# Stereochemical Properties of Two Schiff-Base Transition Metal Complexes and Their Ligand by Using Multiple Chiroptical Spectroscopic Tools and DFT Calculations

**DOI:** 10.3390/molecules28062571

**Published:** 2023-03-12

**Authors:** Guojie Li, Dan Li, Mutasem Alshalalfeh, Joseph Cheramy, Hui Zhang, Yunjie Xu

**Affiliations:** 1 Department of Chemistry, University of Alberta, Edmonton, AB T6G 2G2, Canada; 2 Department of Chemistry, College of Chemistry and Chemical Engineering, Xiamen University, Xiamen 361005, China

**Keywords:** transition metal complexes, conformational landscape, vibrational and electronic circular dichroism, electronic circular dichroism-circularly polarized Raman, exciton chirality method

## Abstract

Two transition metal complexes were synthesized with Ni(II) and Cu(II) using a tetradentate Schiff-base ligand, (*R*,*R*) and (*S*,*S*)-*N*,*N*′-Bis(3,5-di-tert-butylsalicylidene)-1,2-cyclohexanediamine. The stereochemical properties of the ligand and the metal complexes were investigated using a combined experimental and theoretical approach. Multiple spectroscopic techniques, which include IR, vibrational circular dichroism (VCD), UV-Vis and electronic circular dichroism (ECD), as well as Raman and the newly discovered ECD-circularly polarized Raman (i.e., eCP-Raman) spectroscopies were utilized. The good agreement achieved between the experimental and simulated IR, VCD, UV-Vis and ECD spectra of the ligand allowed one to identify the presence of three main ligand conformers in solution, thanks, especially to the high VCD sensitivity to the conformations associated with the tertbutyl groups. The helicity of the metal complexes was identified to be M and P for those with the (*R*,*R*) and (*S*,*S*) ligands, respectively. Furthermore, eCP-Raman measurements were carried out for the two metal complexes under (near) resonance. Their induced solvent chiral Raman features were explained, and the potential application of eCP-Raman was discussed.

## 1. Introduction

Schiff base ligands have attracted much attention in fields ranging from coordination chemistry to organic synthesis to pharmaceutical [1,2]. These ligands are imines with a general formula of R-CH=N-R′ where R and R′ are linear/cyclic alkyl or aryl groups [3]. Thanks to their selectivity and stability, Schiff bases and their transition metal complexes have been increasingly utilized as enantioselective catalysts [4], chiral fluorescent sensors [5] and liquid crystal display devices [6]. A well-designed Schiff base has been considered “a privileged ligand” since it can be easily prepared and used to coordinate with a wide range of different metal centers of various oxidation states [3]. Not only can stereogenic centers or other chirality elements (axes or planes) be introduced into a metal salen complex, the ligands’ backbone and the metal centers can be easily varied and finely tuned in designing a synthesis, including catalytic routes [7]. Moreover, the salen metal complexes themselves can also serve as ligands to further coordinate to a second metal or self-assemble into an oligomer through coordination chromophore and metal substrate [8,9].

Detailed stereochemical properties, such as conformational distribution, ligand chirality and absolute configuration of the metal centers, are of significant importance for further exploration of this fascinating class of chiral molecular systems. Historically, such chirality-related information has been typically obtained using X-ray crystallography, where a high-quality single crystal is needed [10,11]. However, to extract the stereochemical properties of these systems directly in solution, chiral spectroscopic methods, such as electronic circular dichroism (ECD) [12], vibrational circular dichroism (VCD) [13,14,15,16], and vibrational Raman optical activity (ROA) [17] have been utilized. In particular, VCD and ROA, which both exhibit many well-resolved vibrational bands, can provide detailed information not only about the absolute configuration of the system but also about the conformational distributions and intermolecular interactions with solvents [18,19,20] and with the solute itself [21], with the aid of DFT calculations [22].

In this paper, we focus on a chiral tetradentate Schiff-base ligand, (*R*,*R*) and (*S*,*S*)-*N*,*N*′-Bis(3,5-di-tert-butylsalicylidene)-1,2-cyclohexanediamine (salen-chxn) and its complexes with Ni(II) and Cu(II). First, a series of chiroptical spectroscopic measurements were carried out, which include IR, VCD, UV-Vis, ECD, Raman and the newly discovered ECD-circularly polarized Raman, i.e., eCP-Raman [23,24,25,26]. Very recently, eCP-Raman spectroscopy was used to probe chirality recognition by a stereodynamic vanadium probe [27] and to study a series of atropisomeric naphthalenediimides [28]. Therefore, it is of great interest to further explore applications of eCP-Raman spectroscopy with the current systems. Second, applying the recently developed fast conformer-rotamer ensemble sampling tool, CREST [29], systematic conformational searches were performed for both the ligand and the metal complexes. Third, we conducted extensive simulations of the related IR, VCD, UV-Vis, ECD, Raman and eCP-Raman spectra of the ligand and the complexes using the DFT and time-dependent DFT (TDDFT) calculations. As a result, excellent agreements between the experimental and theoretical VCD features were achieved, allowing one to confidently extract stereochemical information about the systems, including ligand conformations and absolute configurations of the center metal and ligands. We also paid attention to the more subtle effects, such as the conformational preference of the tertbutyl (tBu) groups and how they influence the VCD and ECD features. The exciton chirality method, or the coupled oscillator model [30], has been extensively used by chemists to assign absolute configurations based on ECD without any calculations. Its application criteria were very recently reviewed [31]. The coupled oscillator approach has recently been extended to VCD analyses of carbohydrates [32] and peptides [33]. In the current study, the exciton chirality method was applied to both VCD and ECD spectra. We provided some comments about its performance based on the comparison with the associated DFT and TDDFT simulations.

## 2. Results and Discussion

**Scheme 1:**
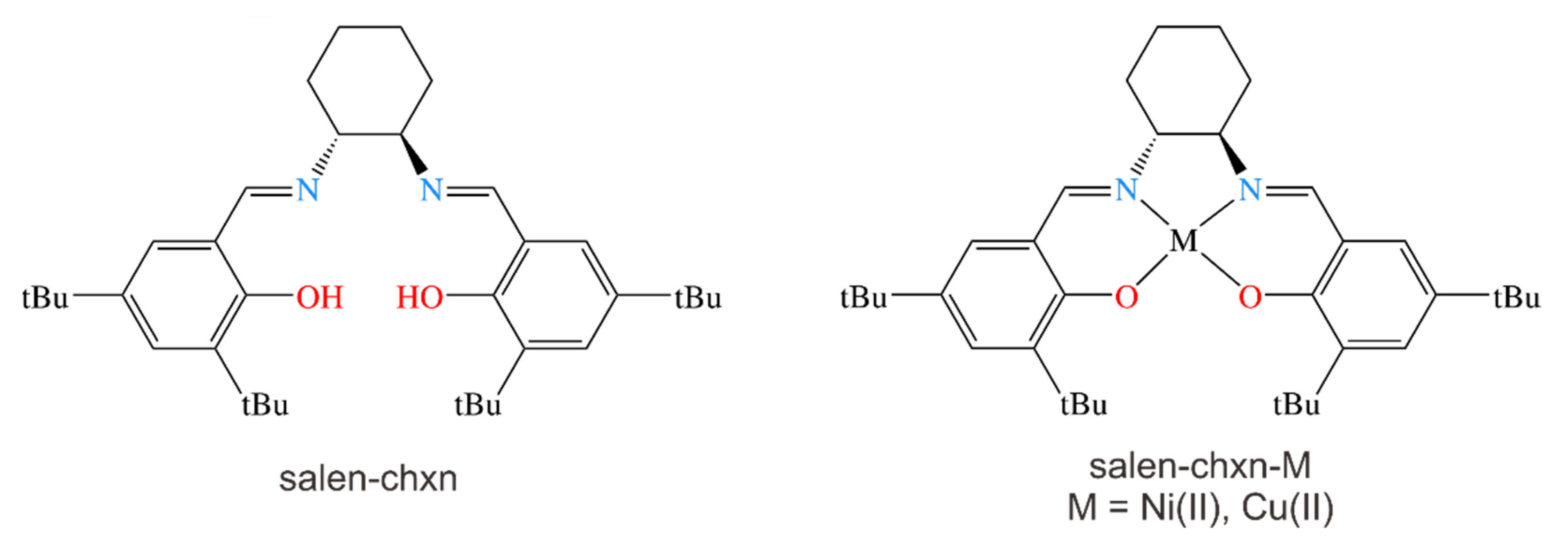
The molecular formula of the (*R*,*R*)-*N*,*N*′-Bis(3,5-di-tert-butylsalicylidene)-1,2-cyclohexanediamine (salen-chxn) ligand and its Ni(II) and Cu(II) complexes, denoted as salen-chxn-Ni(II) and salen-chxn-Cu(II), respectively. tBu indicates the tertbutyl group.

The structural formulas of the Schiff base ligand and its two transition metal complexes are shown in Scheme 1. In the following, we describe the systematic conformational searches of the ligand and the metal complexes and the DFT geometry optimizations done for each compound in Section 2.1. Subsequently, we simulated the IR, VCD, UV-Vis and ECD spectra of the low-energy conformers of salen-chxn. Next, we experimentally compared the final averaged and experimental spectra to extract the preferred conformations in Section 2.2. Then, in Section 2.3, we examined the experimental IR, VCD, UV-Vis and ECD spectra of the salen-chxn-Ni(II) and –Cu(II) complexes and compared them with the simulated ones to extract the helicity of the respective metal complexes. The more subtle effects of the conformations of the tBu groups are also discussed. Overall, the excellent agreements between experiment and theory allow one to conclusively identify the main conformers in the solution. Next, the applications of the exciton chirality model to the VCD and ECD spectra of the ligand and the metal complexes are discussed, and some comments are provided on the applicability of the model in each case in Section 2.4. Finally, in Section 2.5, we describe the eCP-Raman experiments of the two metal complexes under (near) resonance, the analyses of the observed solvent chiral Raman spectra, and the potential applications of eCP-Raman.

While the (*R*,*R*) and (*S*,*S*) enantiomers of the ligand were purchased directly from Sigma-Aldrich, the Ni(II) and Cu(II) complexes were synthesized according to the procedure described in Section 3.1. Therefore, for simplicity, unless otherwise specified, we use the (*R*,*R*)-enantiomer of the ligand and the complexes for all the geometry presentations and spectral simulations in this paper.

### 2.1. Systematic Conformational Searches and Low-Energy Conformers of the Ligand and the Metal Complexes

**Figure 1:**
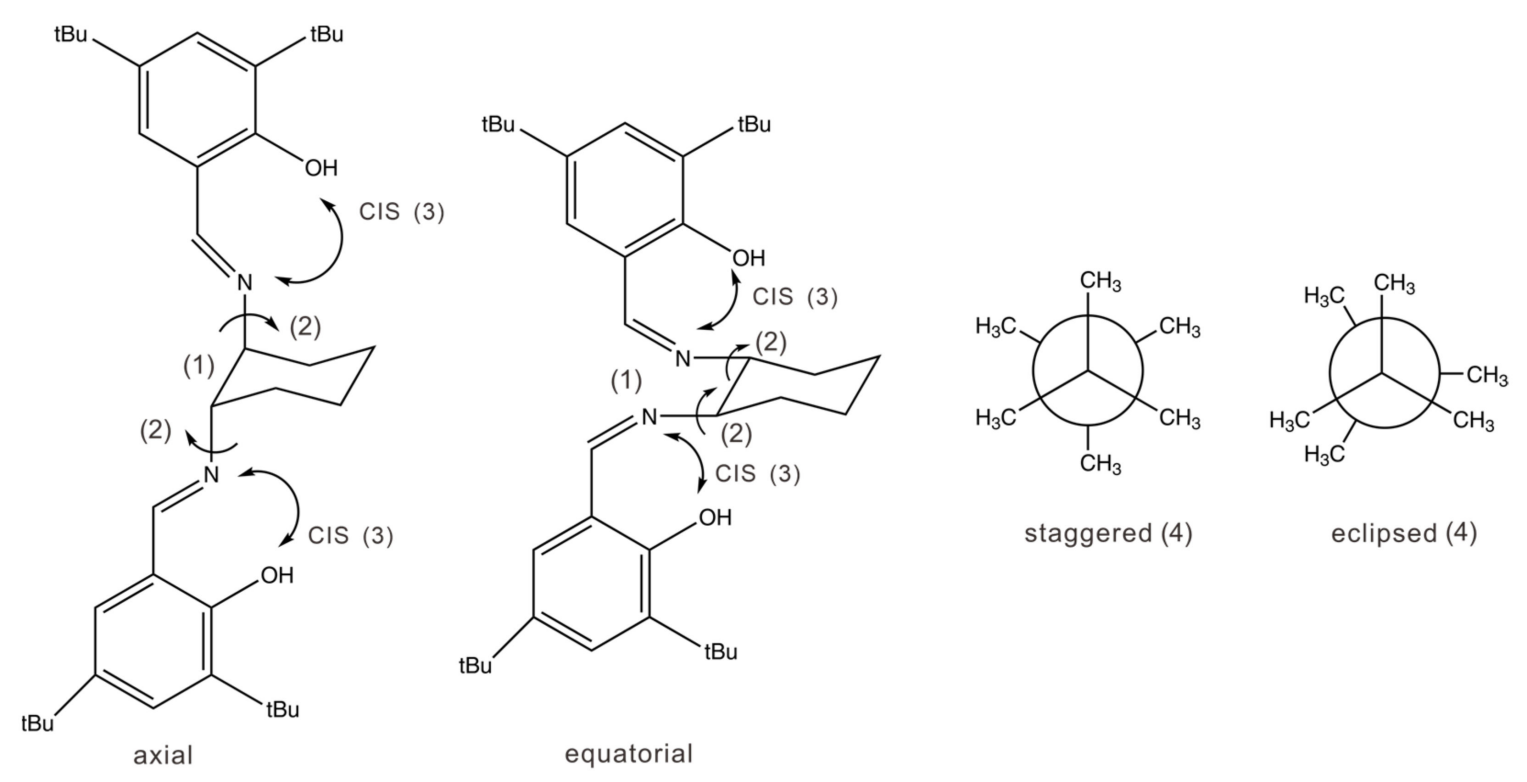
Possible conformations of salen-chxn: (1) the axial and equatorial conformations at cyclohexane; (2) the rotatable N-C_hexane_ bonds are indicated by small arrows; (3) the OH orientation direction pointing to N=C (shown) or away (not shown); (4) the subtle staggered and eclipsed conformations of the tBu groups are also shown on the right side. The eclipsed CH_3_ groups are offset slightly for easy viewing. The tBu groups of the two aromatic branches can take on the staggered–staggered, staggered–eclipsed, and eclipsed–eclipsed conformations.

Based on its chemical formula, the salen-chxn ligand may exhibit a range of conformational flexibilities, as shown in Figure 1. These are associated with the cyclohexane ring conformations, the axial and equatorial positions of the two large substituents, the rotatable motions about the N-C_hexane_ bonds, the OH pointing direction, and finally, the staggered and eclipsed conformations of the two tBu groups in each substituent. Based on the previous VCD studies [14,34] of several related salen ligands with the 1,2-cyclohexanediamine subunit, one would expect the chair conformation of the cyclohexane ring to be significantly more stable than the boat one, while both the axial and equatorial positions support stable conformers. Rather than manually going through these potential ligand conformations, we used the Conformer–Rotamer Ensemble Sampling Tool, i.e., CREST, by Grimme and co-workers [29] for systematic conformational searches. The capability of CREST to generate a nearly complete set of relevant conformations has been extensively benchmarked by gas phase spectroscopic investigations, such as rotational spectroscopic studies of a wide range of molecules and non-covalently bonded clusters [35,36,37] and IR chirality recognition studies of protonated amino acid binary aggregates [38]. For example, CREST was used to identify drastically different binary aggregates of tetrahydro-2-furoic acid observed in a jet-cooled rotational spectroscopic [39] and in a matrix-isolation VCD [40] where very different binary conformers were identified from those in solution [41]. One exception reported recently is the weakly bound heterochiral trimer of propylene oxide [42], where the most stable geometry established experimentally is not a minimum in CREST. Another advantage of CREST is the readily available implicit solvation models for many solvents, which can be used during a conformational search. This capability was utilized in a very recent VCD study of steroids [43].

**Figure 2:**
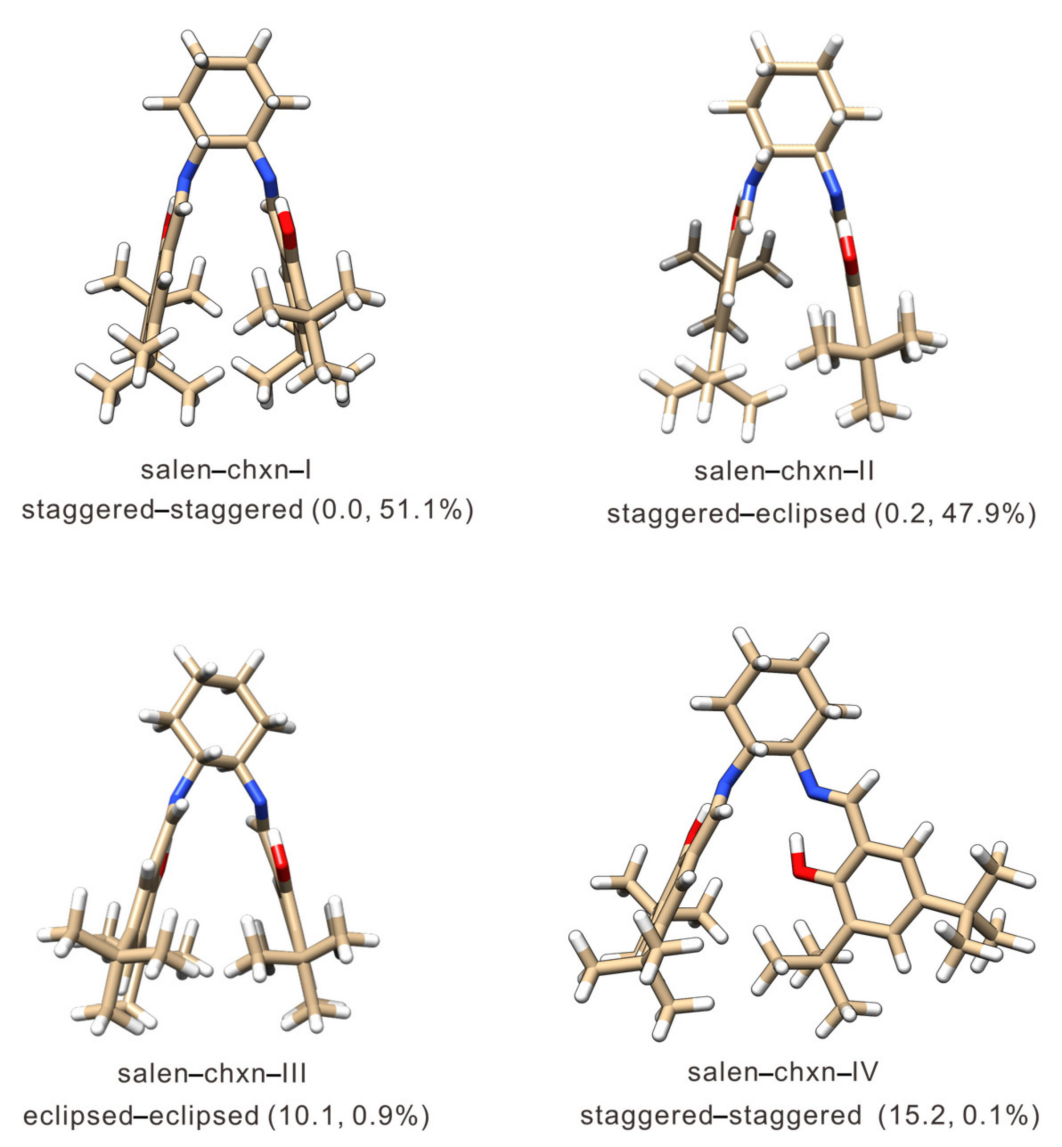
Optimized geometries of the four low energy salen-chxn ligand conformers identified by the CREST search and optimized at the B3LYP-D3BJ/def2-TZVP level of theory with the PCM of CDCl_3_. The relative free energies in kJ mol^−1^ and the Boltzmann percentage abundances at 298 K are provided in the brackets. The blue and red colors represent the nitrogen and oxygen atoms, respectively, whereas the white color is for the hydrogen atoms, and the carbon frame is in the ginger color.

The details of the CREST program and the multitiered approach developed before [38] are described in Section 3, Materials and Methods. In total, 91 ligand geometries were generated after the initial CREST search. The low-level geometry optimization and the subsequent single point energy calculation (see Section 3.2 for details) reduced the number to 48 geometries within an energy window of 20 kJ mol^−1^. Further geometry optimizations were carried out at several different levels of theory, including B3LYP [44,45]/6-31G(d) [46], B3LYP-D3BJ/6-311++G(d,p), and B3LYP-D3BJ/def2-TZVP [47] with the polarizable continuum model (PCM) [48] of CDCl_3_. In addition, the D3 dispersion correction [49,50] with the Becke–Johnson (BJ) damping function [51] was used. The DFT optimization generated four ligand conformers within an energy window of ~15 kJ mol^−1^. The corresponding optimized geometries are depicted in Figure 2, with their relative free energies and their Boltzmann percentage abundances at 298 K.

It is interesting to note that no conformers with the substituents in the axial position were identified by CREST. Even if one constructed an axial conformer as an initial input geometry, the DFT geometry optimization also turned it into an equatorial conformer. Clearly, the axial position does not support a stable conformer. This is different from other related ligands studied before [14], possibly because the substituents in salen-chxn are considerably bulkier. Additionally, only conformers with the OH groups pointing to N=C to form intramolecular H-bonds were identified by CREST within an energy window of 20 kJ mol^−1^. The six-membered H-bonded and benzene rings form a planar structure, bestowing structural rigidity to the main frame of the salen-chxn ligand. As seen in Figure 2, the relative orientation of these two planar substituent structures leads to different types of conformers. The most stable type (Type I) has these two planar substituents stretching out with the OH group up in one substituent and down in the other. In contrast, other relative orientations, for example, with the two planes roughly perpendicular to each other (Type II), are more than 15 kJ mol^−1^ less stable. Overall, the three most stable Type I conformers take on the staggered–staggered (global minimum), staggered–eclipsed, and eclipsed–eclipsed tBu arrangements, while the next less stable conformer is Type II with the staggered–staggered tBu arrangement (see Figure 2).

**Figure 3:**
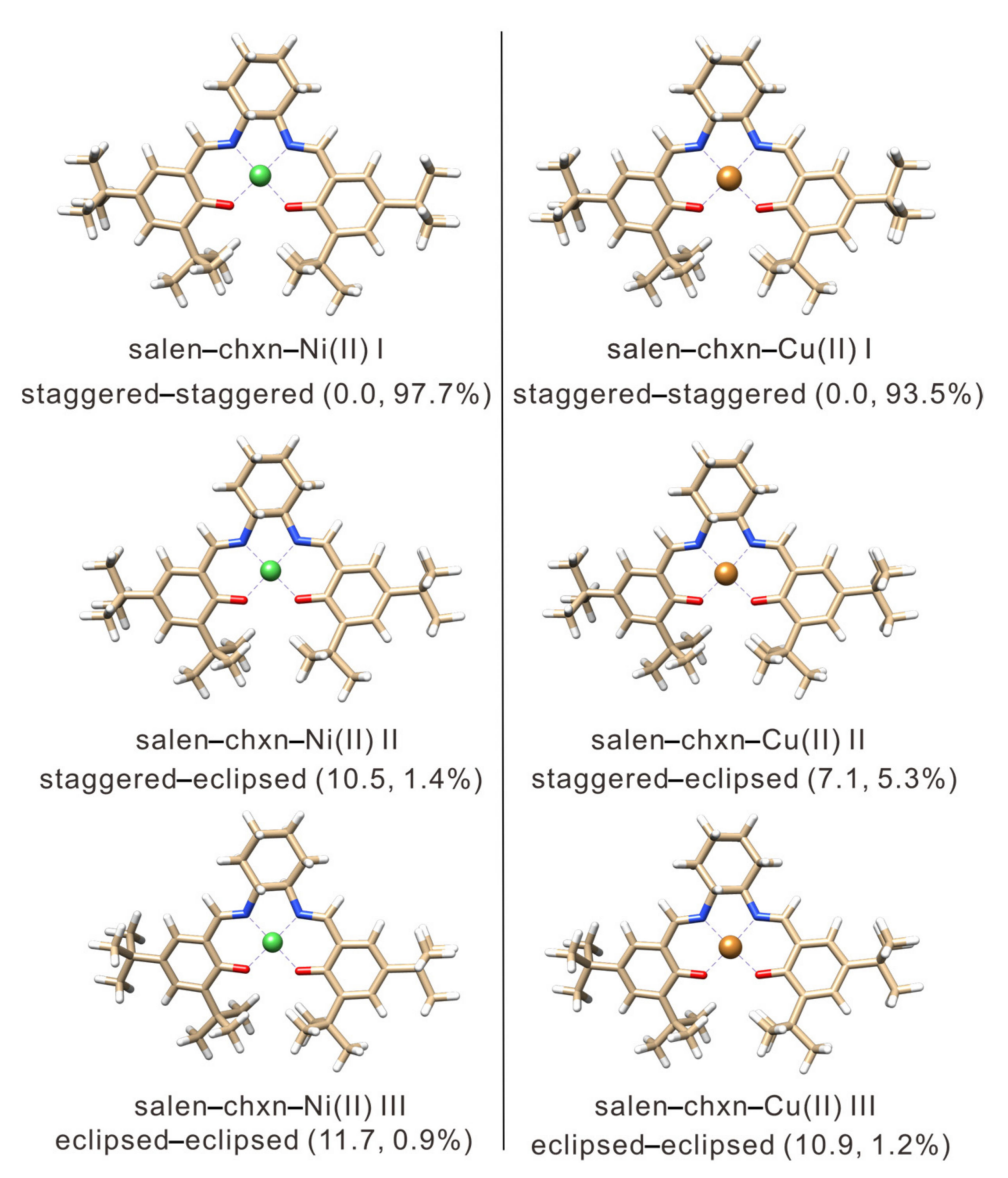
The optimized geometries of the salen-chxn-Ni(II) and salen-chxn-Cu(II) conformers at the B3LYP-D3BJ/6-311++G(d,p) level of theory with the PCM of CDCl_3_. The relative free energies in kJ mol^−1^ and the Boltzmann percentage abundances at 298 K are provided in the brackets. The blue and red colors represent the nitrogen and oxygen atoms, respectively, whereas the white color is for the hydrogen atoms, and the carbon frame is in the ginger color. The green and gold colors represent the nickel and copper atoms, respectively.

For the salen-chxn-Ni(II) and salen-chxn-Cu(II) complexes, the same conformational searches as described above were conducted. However, the coordination with the metal centers makes these systems more rigid than the salen-chxn ligand, and only nine candidates were identified. The geometry optimizations were performed at the B3LYP-D3BJ/6-311++G(d,p) level and the combined B3LYP-D3BJ/def2-TZVP (for all atoms except the metal atom) + LANL2DZ (Los Alamos National Laboratory 2 double-ζ [52], for Ni and Cu) level. The DFT calculations led to three conformers associated with the different tBu arrangements. Consistent results were obtained at both levels of theory. The optimized geometries of the three conformers for Ni and three for Cu obtained are summarized in Figure 3, with their free energies and Boltzmann factors at 298 K.

### 2.2. Experimental and Simulated IR, VCD, UV-Vis and ECD Spectra of the Salen-Chxn Ligand

**Figure 4:**
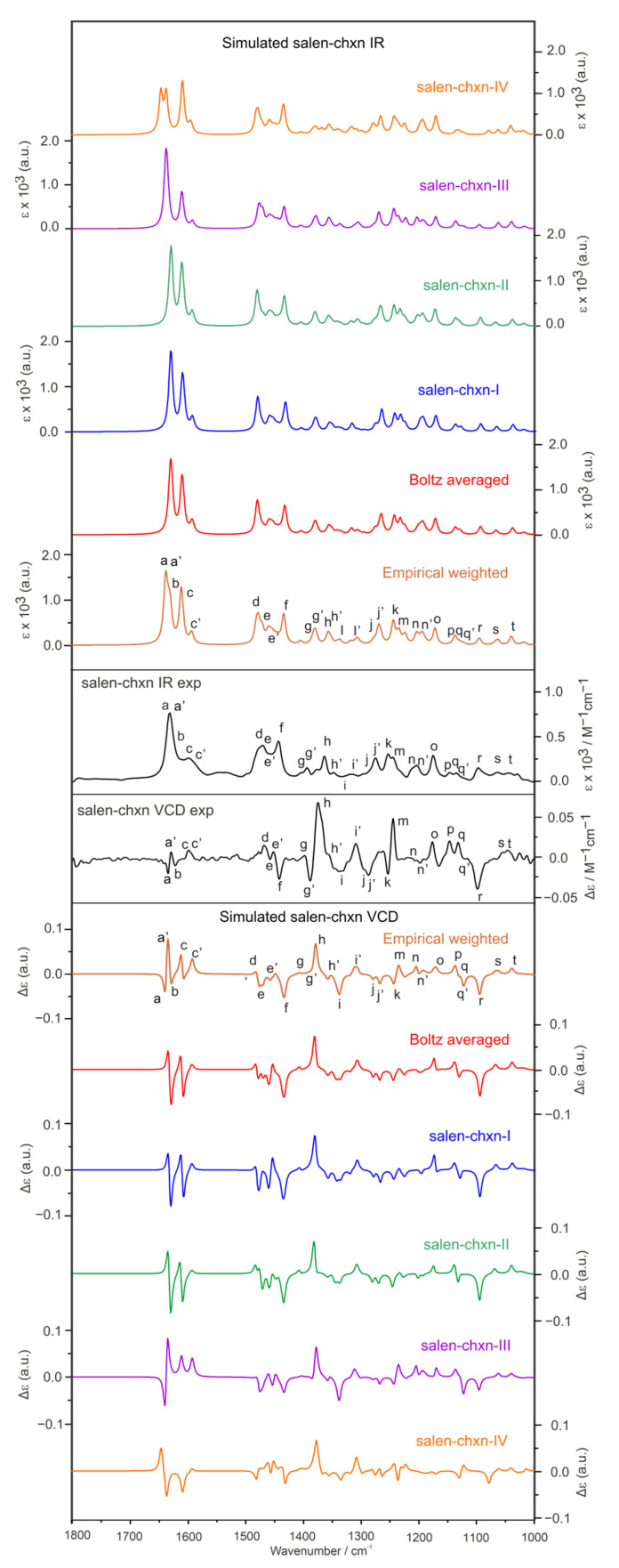
The simulated individual IR and VCD spectra of the four most stable salen-chxn conformers and the related Boltzmann averaged IR and VCD spectra at the B3LYP-D3BJ/def2-TZVP/PCM(CDCl_3_) level of theory at 298 K. The experimental and empirically weighted IR and VCD spectra are also provided. The experimental units are on the right side. The main IR and VCD band features are labelled “a” to “t” to aid the comparison.

The individual conformer IR and VCD spectra of the four most stable salen-chxn conformers are depicted in Figure 4, together with the Boltzmann averaged and the experimental IR and VCD spectra of the ligand in CDCl_3_ solution. In terms of the IR spectra, those of salen-chxn-I (staggered–staggered) and -II (staggered–eclipsed) look almost identical, whereas those of salen-chxn-III (eclipsed–eclipsed) and -IV (Type II staggered–staggered) demonstrate some noticeable differences. In general, it is easy to correlate the experimental IR bands with the calculated ones, and all the visible features are labelled with letters “a” to “t” for easy recognition. For example, the highest wavenumber bands of salen-chxn-III, i.e., those labelled as a (antisymmetric) and a’ (symmetric) for the C=N stretching modes, are blue-shifted by ~7 cm^−1^ compared to those of -I and -II, and the relative intensity of b of -III is somewhat weaker than those of -I and -II. For salen-chxn-IV, the C=N and aromatic ring C=C stretching region exhibits a much different pattern in terms of spacing and relative intensity when compared to those of -I, -II and -III.

**Figure 5:**
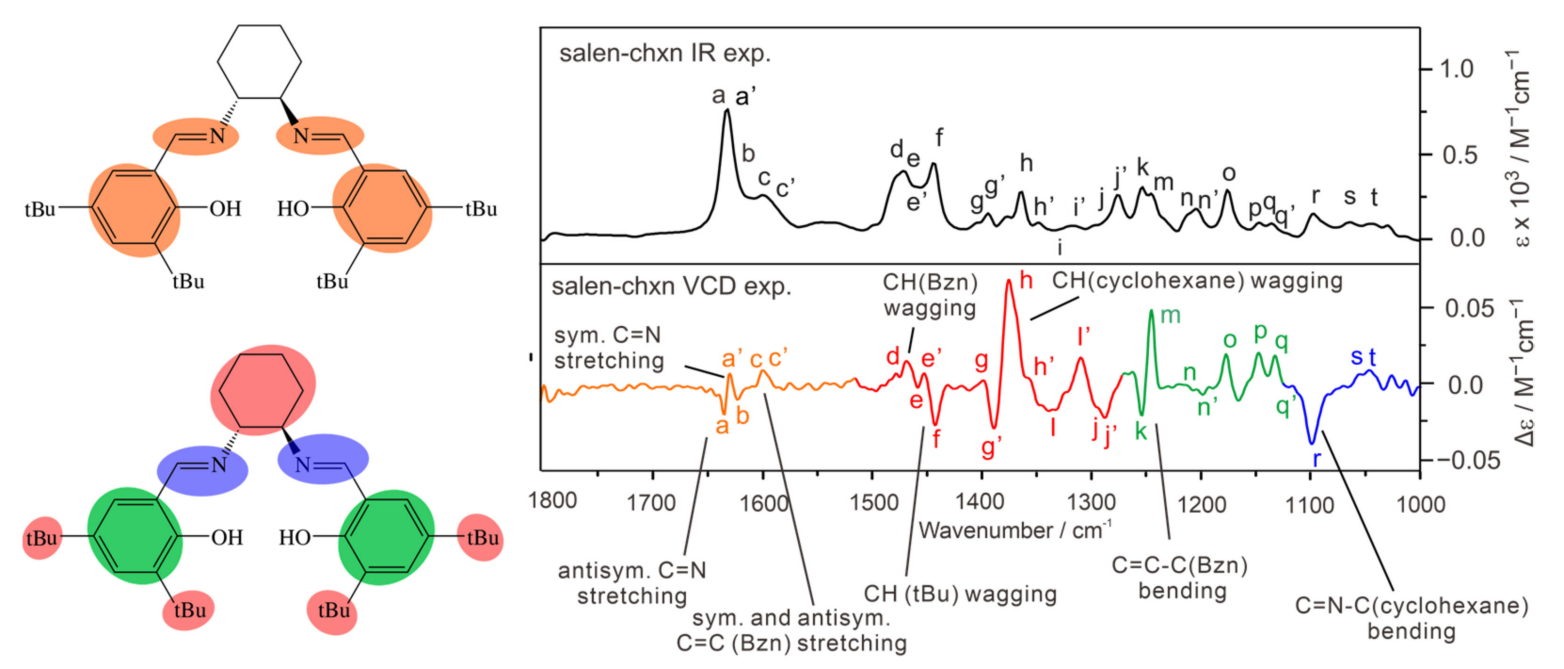
The general assignment of the IR and VCD bands of salen-chxn. The main IR and VCD band features are labelled “a” to “t” to aid the comparison as in Figure 4.

To facilitate vibrational discussion, the experimental IR band assignments are provided in Figure 5, based mainly on the calculated IR spectrum of the dominant conformer, salen-chxn-III and -I (vide infra). As mentioned above, band a and a’ correspond to the antisymmetric and symmetric C=N stretches, respectively. While the unresolved b band contains the symmetric and antisymmetric aromatic ring C=C stretching bands, the c and c’ are the symmetric and antisymmetric wagging motions of the CHs of the aromatic ring coupled with the OHs, respectively.

As in the case of their IR spectra, the VCD spectra of salen-chxn-I and -II are very similar, except in the 1420–1500 cm^−1^ region (bands d to f) where some minor differences are noted, mostly related to the C-H wagging motions from the tBu groups and the benzene rings. While salen-chxn-III and -IV offer similar main VCD features in the region below 1580 cm^−1^, they exhibit drastically different VCD spectra from those of -I and -II in the above 1580 cm^−1^ region. Compared to the experimental VCD features, it is obvious that only salen-chxn-III shows the negative/positive VCD signs (high to low cm^−1^) of the C=N stretching bands a and a’ in the ~1635 cm^−1^ region, which matches the experimental ones. Salen-chxn-I, -II and -IV, on the other hand, all offer wrong VCD sign patterns. In the 1245 cm^−1^ region, salen-chxn-III exhibits a strong positive VCD feature, which matches the experimental band m intensity. In contrast, the other three conformers offer a relatively weak VCD feature at this position or not at all.

Overall, salen-chxn-III offers the best agreement with the experimental IR and VCD features, especially in the above 1580 cm^−1^ region and around 1250 cm^−1^. At the same time, salen-chxn-I and -II provides somewhat better agreements in the 1300 cm^−1^ region centered on feature i and in the 1100 cm^−1^ region centered on feature r. In general, the predicted VCD features of salen-chxn-IV do not offer any good agreement with the experimental ones in all the main regions discussed above. Clearly, a combination of substantial salen-chxn-III and some amount of salen-chxn-I and -II would be needed to account for the observed IR and VCD features. Therefore, we also included the empirically weighted IR and VCD spectra in Figure 4, where empirical percentage abundances of 60%, 20% and 20% were assigned to salen-chxn-III, -I and -II, respectively. The empirical abundances are somewhat different from the theoretical Boltzmann percentages, which overly emphasize the contribution of salen-chxn-I and -II and downplay the importance of salen-chxn-III. Nevertheless, based on the good agreement between the experimental and empirically weighted theoretical IR and especially VCD spectra, we can conclude that salen-chxn-I, -II and -III are all important conformers in solution, with -III being the most abundant one.

With regard to the three tBu conformations, one may initially assume that the staggered arrangement would be noticeably more stable than the eclipsed one because of the Newman projections shown in Figure 2. But a closer examination reveals that the two tBu groups are so far apart that the closest H···H distance is still over 3.6 Å, very unlikely to make a noticeable difference in their relative free energy. Actually, the closest H···H distances are those between the tBu group and the aromatic ring. They are about 2.1 Å, again not likely to make much difference in the relative energy. Indeed, the DFT free energy difference for salen-chxn-I (staggered–staggered) and -II (staggered–eclipsed) is quite small, i.e., <0.2 kJ mol^−1^. It was puzzling initially why salen-chxn-III (eclipsed–eclipsed) is so much less stable than salen-chxn-II (staggered–eclipsed) or salen-chxn-I (staggered–staggered). A further examination revealed that to accommodate the eclipsed–eclipsed tBu arrangement, the tBu groups in one aromatic branch become too close to the tBu groups in another branch, generating much steric tension and pushing the two aromatic branches a bit further apart. This change, although subtle, can be seen visually in the changes of IR and VCD bands above 1400 cm^−1^, especially those associated with the C=N stretches and aromatic ring C=C stretches. This is also reflected strongly in the relative free energies with salen-chxn-III predicted to be ~10 kJ mol^−1^ less stable.

Figure S1 (Supplementary Materials) provides the bond critical points related to the non-covalent interactions based on the Quantum Theory of Atoms in Molecules (QTAIM) [53] analysis. As one can see, salen-chxn-I has one more cage critical point than -III, further stabilizing -I. On the other hand, comparing the experimental IR and, in particular, the VCD features with the simulated ones strongly indicates that all three conformers, i.e., salen-chxn-I, -II and -III, are all present dominantly in the solution. Therefore, one may speculate that the additional cage critical point in salen-chxn-I may limit its interactions with the solvent. In salen-chxn-III, on the other hand, the aromatic rings may be more ready to accommodate some solvent molecules, making it more stable in the solution through some explicit solute–solvent interactions. We also note that the single point energy calculations at the DLPNO-CCSD(T) [54]/cc-pVDZ level using ORCA [55] reduce the relative free energy difference between salen-chxn-I and -III to 7.1 kJ mol^−1^, suggesting that the true energy difference may be overestimated at the DFT level. Please note that the relative Gibbs energy corrections were taken directly from the DFT calculations, and the PCM of CDCl3 was included in the single point calculations.

**Figure 6:**
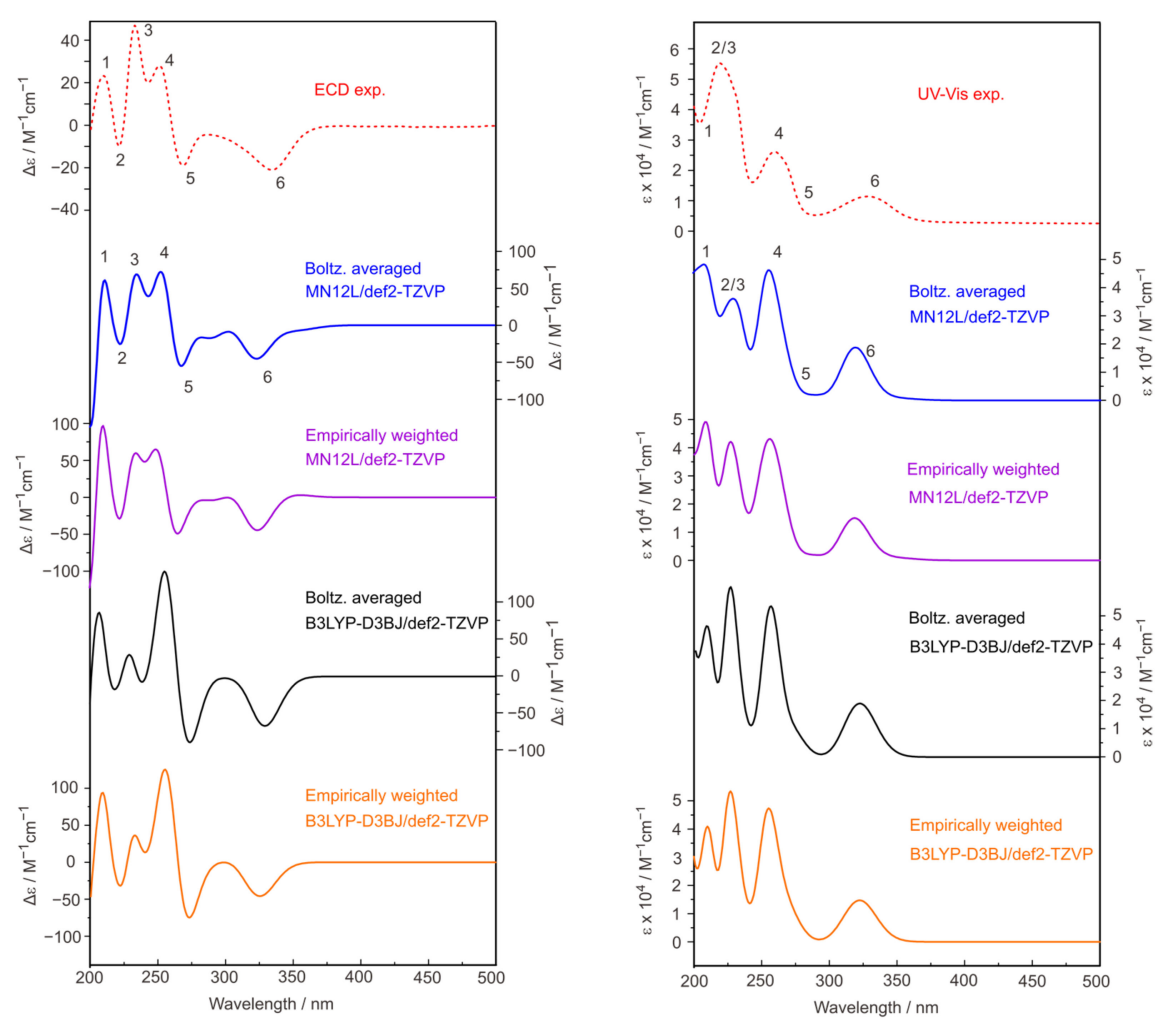
Comparison of the experimental and theoretical UV-Vis and ECD spectra of salen-chxn in acetonitrile solution. The first 200 electronic states were included in the calculations at the two levels of theory indicated. For the empirically weighted spectra, we used the same weights derived from the IR and VCD comparison. The main UV-Vis and ECD bands are labelled as “1” to “6” to aid the comparison.

The experimental UV-Vis and ECD spectra of salen-chxn in acetonitrile (ACN) are provided in Figure 6. The UV-Vis and ECD spectra of the three dominant conformers derived from the IR and VCD analyses discussed above were calculated at two different levels of theory: B3LYP-D3BJ/def2-TZVP which was used for the IR and VCD calculations above, and MN12L/def2-TZVP. The individual UV-Vis and ECD spectra of salen-chxn-I, -II and -III are provided in Figure S2, while the related Boltzmann weighted spectra are also depicted in Figure 6. In addition, the empirically weighted UV-Vis and ECD spectra using the percentage abundances obtained from the IR and VCD analyses are also shown in Figure 6.

It is clear that the UV-Vis and ECD spectra of these three conformers look very similar. Not surprisingly, the Boltzmann and empirically weighted UV-Vis and ECD spectra look very similar because the UV-Vis and ECD features are much less sensitive to the subtle tBu conformations. Taking advantage of better resolution with ECD, the experimental peaks at 209 nm, 221 nm, 233 nm, 250 nm, 266 nm and 330 nm can be tentatively assigned to the calculated ones labelled 1 to 6. Overall, the simulated UV-Vis and ECD spectra at the MN12L level provide a better agreement with the experimental ones than the B3LYP level.

### 2.3. Experimental and Simulated IR, VCD, UV-Vis and ECD Spectra of the Salen-Chxn-Ni(II) and Cu(II) Complexes

**Figure 7:**
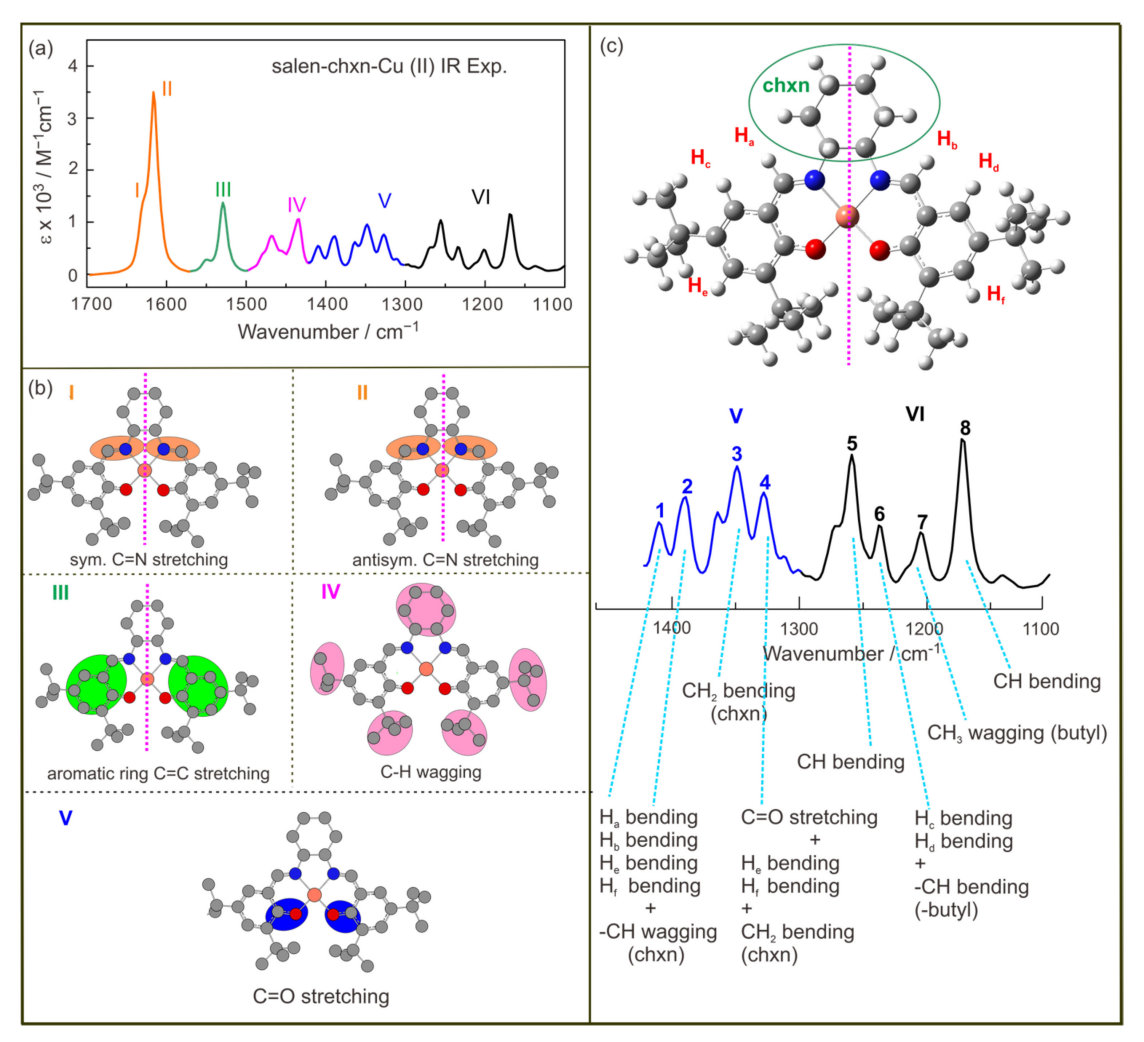
(**a**) The IR spectrum of salen-chxn-Cu(II) in the 1700–1100 cm^−1^ region is divided into six parts from I to VI. (**b**) The molecular parts related to I to V are coloured. (**c**) The main vibrational modes of salen-chxn-Cu(II) in the 1450–1100 cm^−1^ region. The main IR bands in the 1100–1450 cm^−1^ region are labelled as “1” to “8” to aid the discussion.

Since the IR features of these two complexes are quite similar, we first focus on the IR band assignment and the comparison of the IR bands of the complexes with the ligand, using salen-chxn-Cu(II) as an example. One goal here is to facilitate an in-depth understanding of the appearance of several strong bisignate VCD couplets compared to the ligand. The band assignments are given in Figure 7, based on the theoretical simulation (vide infra). The region of interest is roughly divided into six regions, namely I-VI, associated with the vibrational motions centered at different parts of the complex, as shown in Figure 7.

The shoulder I is mainly associated with the C=N symmetric stretch, while II is dominated by the C=N antisymmetric stretch with considerable contributions from the aromatic ring C=C stretches. In comparison to the ligand, the antisymmetric C=N stretch is red-shifted, whereas the aromatic ring C=C stretches appear to be slightly blue-shifted. Region III contains mostly aromatic ring C-H wagging motions, demonstrating a noticeably strong intensity compared to the somewhat related vibrational modes in the ligand, i.e., band c and c’, which are of much lower intensity and blue-shifted greatly. While region IV is mainly related to the cyclohexane CH_2_ and tBu CH_3_ motions, region V contains the symmetric and antisymmetric C-O stretches. The rest of the low wavenumber features are grouped into VI, and their more detailed assignments are provided in Figure 7.

**Figure 8:**
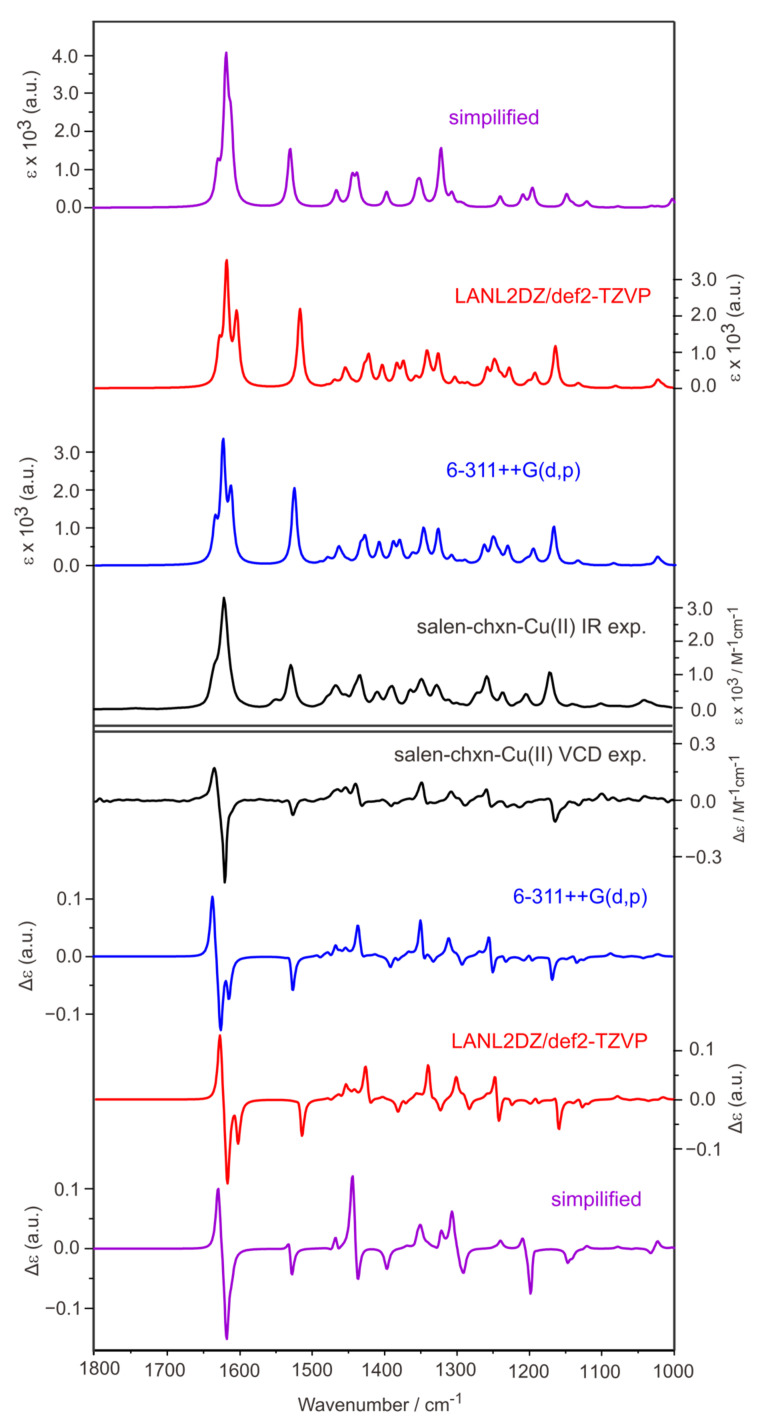
Comparison of the experimental IR (**top**) and VCD (**bottom**) spectra of salen-chxn-Cu(II) with the corresponding calculated spectra in CDCl_3_ solution. The levels of theory used are B3LYP-D3BJ/6-311++G(d,p) and B3LYP-D3BJ/def2-TZVP+LANL2DZ(for Cu) with the PCM of chloroform. The simplified calculations were done by replacing tBu with an H atom.

As described in Section 2.1, three low-energy conformers were identified for each metal complex. The Boltzmann weighted simulated IR and VCD spectra of salen-chxn-Cu(II) at the B3LYP-D3BJ/6-311++G(d,p) and B3LYP-D3BJ/def2-TZVP+LANL2DZ(for Cu) levels are compared with the experimental ones in Figure 8. The associated individual conformer IR and VCD spectra at the B3LYP-D3BJ/6-311++G(d,p) level are shown in Figure S3. It is interesting to note that both IR and VCD spectra of these three conformers look very similar, unlike in the case of the ligand itself. This means that the IR and VCD spectra do not offer discrimination among these tBu conformers.

Generally, excellent agreements were achieved between the experimental and theoretical IR and VCD spectra of salen-chxn-Cu(II). We note that all visible IR and VCD experimental features are reproduced theoretically. The strongest VCD features in the Cu(II) complex are the negative/positive (from low to high cm^−1^) bisignate features in regions I and II. These bisignate features are associated with the antisymmetric and symmetric C=N stretches in the Cu(II) complex based on the simulation, a point which will be further explored in Section 2.4. On the other hand, the two aromatic rings C=C stretches in region II are not strongly coupled. In fact, the GaussView visualization shows them as mainly located at the right and the left branches separately. This situation leads to an overall negative VCD which is not well resolved from the negative wing of the aforementioned bisignate feature.

**Figure 9:**
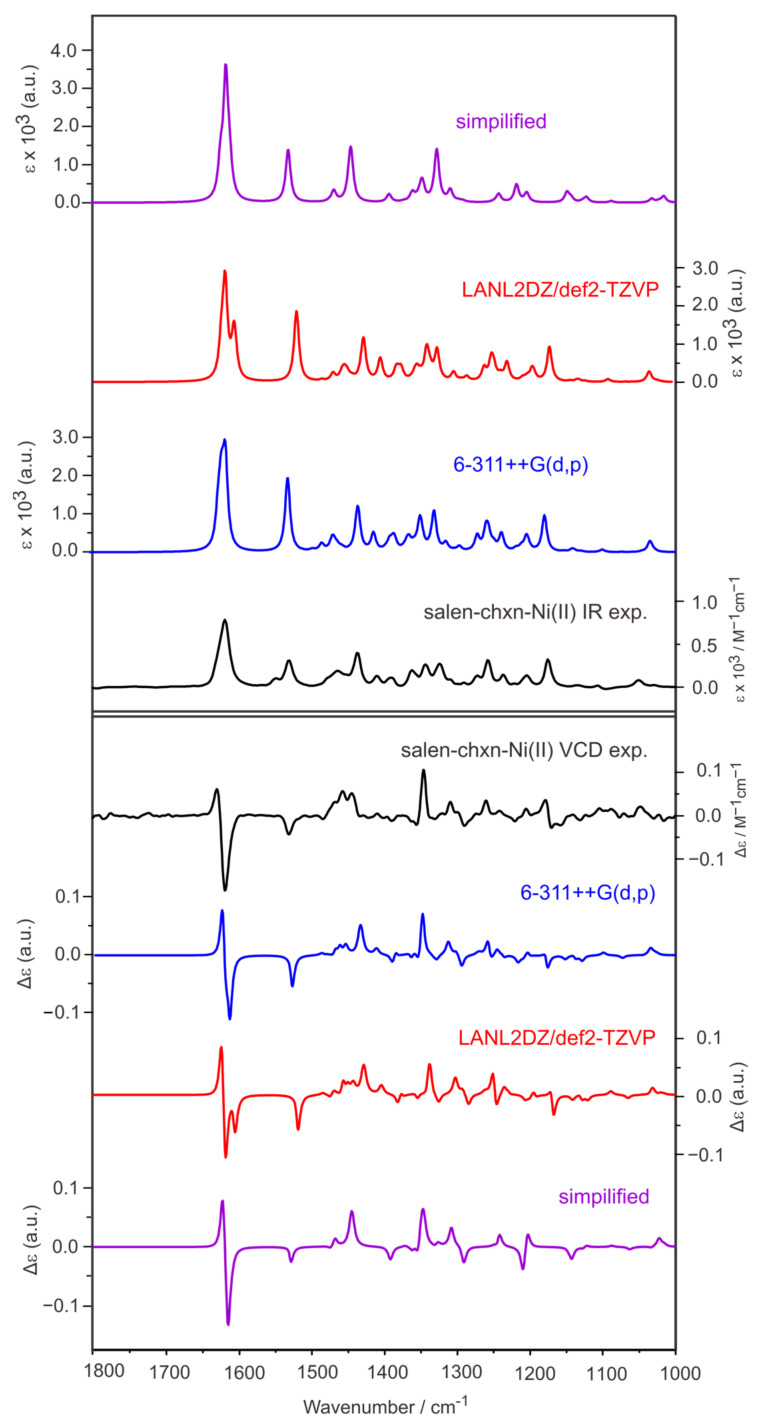
Comparison of the experimental IR (**top**) and VCD (**bottom**) spectra of salen-chxn-Ni(II) with the corresponding calculated spectra in CDCl_3_ solution. The levels of theory used are B3LYP-D3BJ/6-311++G(d,p) and B3LYP-D3BJ/def2-TZVP + LANL2DZ(for Ni) with the PCM of chloroform. The simplified calculations were done by replacing tBu with an H atom.

Parallel calculations and comparisons were also performed for the salen-chxn-Ni(II) complex. While the Boltzmann-weighted simulated IR and VCD spectra of salen-chxn-Ni(II) are compared with the experimental ones in Figure 9, the associated individual conformer IR and VCD spectra are also shown in Figure S3. Again, excellent agreements between theory and experiment have been achieved, and neither IR nor VCD features are sensitive to the different conformations due to the tBu arrangements.

As discussed above, the simulated IR and VCD spectra agree very well with the experimental features for both the Ni(II) and Cu(II) complexes, supporting that the most stable conformation predicted for each complex (see Figure 3) is the main species in solution. In contrast to the ligand case where the staggered–staggered (20%), staggered–eclipsed (20%), and eclipsed–eclipsed (60%) salen-chxn conformers all contribute significantly to the observed spectra, the staggered–staggered conformation prevails with ~97% and 93% abundances for the salen-chxn-Ni(II) and -Cu(II) complexes in solution, respectively. We do acknowledge that the IR and VCD features are not very sensitive to the tBu conformations, unlike in the case of the ligand.

Another goal of the current study is to explore the possibility of speeding up the calculations by replacing each tBu group with an H atom and verifying if the simplified calculations can theoretically capture all major experimental IR and VCD features. The simplified calculation was performed at the B3LYP-D3BJ/6-31G(d,p) (for C and H atoms) + 6-311++G(2d,p) (for Ni or Cu, N and O atoms) level. The results are also presented in Figure 8 and Figure 9 for the Cu(II) and Ni(II) complexes, respectively. It is interesting to note that major IR and VCD features in the above 1500 cm^−1^ region were well reproduced. In contrast, those medium strength bands in the below 1500 cm^−1^ region for the Ni(II) and Cu(II) complexes were not captured accurately. This is not surprising because these specific bands below 1500 cm^−1^ can be largely assigned to the CH_3_ motions of the tBu functional groups as well as the CHs associated with the cyclohexane ring. Therefore, if one’s main focus is the absolute configuration of the metal complexes, it is enough to do the simplified calculations. On the other hand, the complete calculations allow detailed assignment of all visible vibrational and VCD bands, greatly boosting the confidence of conformational identification.

Another key point of interest is the absolute configuration of the chiral metal centers, i.e., the helicity of the metal complexes [14]. The dihedral angle θ which defines the specific helicity preference, is the dihedral angle between the O1-Metal-N1 and the O2-Metal-N2 planes shown in Figure S4, where O1 and N1 belong to one aromatic branch, and O2 and N2 belong to another. These angle values are 3.7 and 9.0 degrees for the Ni(II) and Cu(II) complexes, respectively. Since these two metal complexes exhibit nearly square planar geometries, one may speculate that the M or P helicity would be possible because only a small change in the dihedral angle to the opposite direction would be enough for the helicity switch. In a previous study of several Ni(II) complexes which are in a distorted square planar N_2_O_2_-coordination sphere, a diastereomeric equilibrium was detected, with a strong preference of the Λ (i.e., M) over Δ (i.e., P) metal chirality for the R-ligands [56]. The preference for Δ or Λ was shown to be tunable by using different solvents in the case of a tris(diamine)nickel(II) complex [57]. 

Interestingly, the situation is quite different for the nearly square planar Ni(II) and Cu(II) complexes with the salen-chxn ligands. No P helicity geometry candidates were identified in the CREST searches within an energy window as high as 60 kJ mol^−1^. To further confirm the absence of stable P helicity geometries, we also constructed several Ni (II) geometries with the P helicity as the starting geometries. All of them reverted to the M helicity with the DFT geometry optimization. It appears that the cyclohexane ring, which connects the two nitrogen atoms of the ligand, strongly influences the outcome of the possible diastereomers with the (*R*,*R*) ligands, i.e., only the M helicity diastereomers are stable for the (*R*,*R*)-salen-chxn-Ni(II) and Cu(II) complexes. This calculation result, combined with the excellent agreement between the experimental and theoretical IR and VCD of the (*R*,*R*)-salen-chxn-Ni(II) and Cu(II) complexes, allows one to unequivocally state that the dominant conformers are with the M metal chirality. This conclusion is similar to that drawn for other nearly square planar Schiff base transition metal complexes with similar ligands, for example, the bis(pyrrol-2-ylmethyleneamine)-cyclohexane-Ni(II), Cu(II), Pd(II) and Pt(II) complexes reported previously [14].

**Figure 10:**
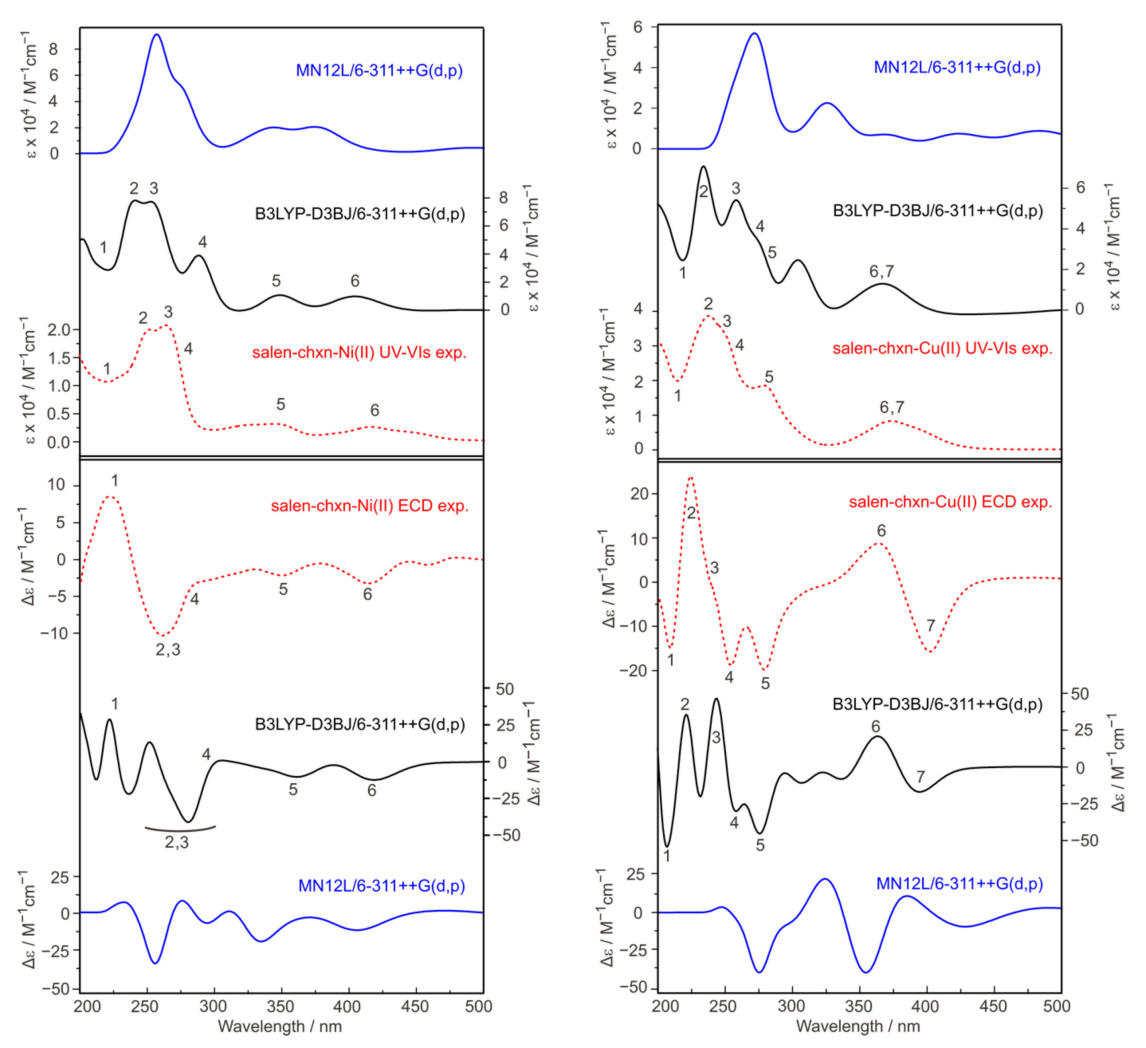
Comparison of the experimental and simulated UV-Vis (**top**) and ECD (**bottom**) spectra of salen-chxn-Ni(II) and salen-chxn-Cu(II) at two different levels of theory. In total, 250 electronic states were included in the calculation. The main UV-Vis and ECD bands of salen-chxn-Ni(II) and salen-chxn-Cu(II) are labelled as “1” to “6” and “1” to “7”, respectively, to aid the comparison.

The experimental UV-Vis and ECD spectra of the salen-chxn-Ni(II) and salen-chxn-Cu(II) are depicted in Figure 10. From the IR and VCD analyses, we know that, by far, the dominant conformation is staggered–staggered. Therefore we carried out TDDFT calculations for the most stable salen-chxn-Ni(II) and salen-chxn-Cu(II) conformers with 97% and 93% abundances, respectively. The resulting UV-Vis and ECD spectra are summarized in Figure 10 as well. To facilitate the comparison, noticeable experimental UV-Vis and ECD bands are numbered 1 to 6 or 7, and the corresponding band assignments in the calculated spectra are also numbered accordingly. Because of the broad nature of the spectral features in the UV-Vis region, the assignments are less conclusive in comparison to the IR and VCD assignments. At the same time, the calculated UV-Vis and ECD spectra at the two different levels of theory differ noticeably, highlighting the challenges in modelling transition metal complexes. Interestingly, the B3LYP-D3BJ/6-311++G(d,p) provides considerably better agreement with the experiment for both metal complexes than MN12L/6-311++G(d,p). It is known that the performance of different combinations of DFT functionals and basis sets usually need to be tested for the excited state calculations of transition metal complexes because of the associated challenges [15]. The good agreement provided by the B3LYP-D3BJ/6-311++G(d,p) calculations allowed one to assign most of the major features observed. Overall, one could confidently state that the helicity of the metal centers, i.e., Ni(II) and Cu(II), are indeed M based on the ECD analyses, the same conclusion derived from the corresponding VCD analyses.

### 2.4. Applications of the Exciton Chirality Method to the VCD and ECD Spectra of the Complexes

The exciton chirality method for ECD spectroscopy was recently comprehensively reviewed by Pescitelli [31]. The author outlined three main prerequisites for correctly applying the exciton chirality method. These include: (i) knowing the conformational distribution of the molecular target; (ii) knowing the corresponding direction of the electric transition moments; and (iii) assuming the observed ECD signals to be dominated by the exciton coupling mechanism. Below we first discuss whether it is suitable to apply the exciton chirality method to the ECD spectra of the ligand and the two complexes and use the theoretical calculations in the current study to rationalize the conclusion. Second, we extend the exciton chirality method application to the VCD spectra of the three compounds studies.

For the salen-chxn ligand, it is unsuitable for applying the exciton chirality method to interpret the resulting ECD features because the system does not fulfil criteria (ii) and (iii). There are multiple chromophores in the UV-Vis region, and most bands appear to be severely overlapped, making it difficult to identify the exciton couplets. The situation for the two metal complexes seems somewhat promising at first. In the experimental ECD spectrum salen-chxn-Ni(II), one strong negative Cotton couplet is in the region below 300 nm. A clear negative Cotton couplet centered at about 385 nm is present in the salen-chxn-Cu(II) ECD spectrum. On the other hand, as one can see in Figure 10, the ECD spectra of these two metal complexes look very different even though they share many similarities (although Cu(II) is an open shell and Ni(II) is a closed shell), making it difficult to fulfill requirement (ii). To illustrate this difficulty, the contributions of the individual electronic transitions to the UV-Vis and ECD spectra are provided in Figure S5. It is clear that the negative Cotton couplet contains some obvious contributions by multiple electronic transitions rather than an obvious pair of coupled transitions. Salen-chxn-Cu(II) suffers a similar issue. Some of these difficulties associated with applying the exciton chirality method to the salen Ni(II) complexes were also alluded to in a previous publication [58].

For the salen-chxn ligand, applying the exciton chirality method to interpret the resulting VCD features is unsuitable because none of the three criteria are fulfilled. While salen-chxn-I, -II and -III all have similar arrangements of the two aromatic branches, the resulting VCD features are sensitive to more subtle differences, such as the orientations of the tBu groups. Second, there are no obvious observed VCD features dominated by the exciton coupling mechanism.

**Figure 11:**
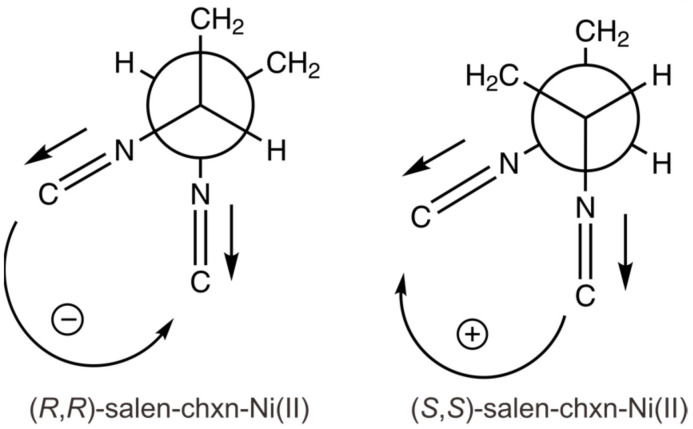
The two coupled C=N stretching transition dipole moments are indicated for the (*R*,*R*)-salen-chxn-Ni(II) and (*S*,*S*)-salen-chxn-Ni(II), providing the negative and positive exciton coupling couplets, respectively.

The situation improves with the two metal complexes. First of all, both of their observed experimental VCD features are dominated by a negative VCD couplet in the above 1580 cm^−1^ region where the lower wavenumber band shows a negative sign. Second, the bisignate VCD couplets of salen-chxn-Ni(II) and -Cu(II) are related to the symmetric and antisymmetric C=N stretches, whose vibrational electric transition moment directions are well defined, as shown in Figure 11. Second, only one main conformer was identified for each metal complex, or at least the minor tBu arrangements do not affect the relative orientation of the two aromatic branches, thanks to the rigidity introduced by the coordination bonding to a central metal atom. Following the VCD exciton chirality method proposed by Taniguchi and Monde [59], the method indeed predicted a negative Cotton effect for (*R*,*R*)-salen-chxn-Ni(II) (Figure 11), marked by a counter-clockwise rotation of the C=N groups. The same scheme can be applied to salen-chxn-Cu(II).

### 2.5. Experimental and Simulated eCP-Raman Spectra of Salen-Chxn-Ni(II) and Salen-Chxn-Cu(II)

Recently, it was discovered that when a chiral compound, such as a transition metal complex, is under (near) resonance, the chiral Raman measurement carried out using a ROA spectrometer may contain significant eCP-Raman contributions in addition to resonance ROA features. However, since there have been very few examples reported of the newly discovered eCP-Raman spectroscopy, we conducted further Raman and eCP-Raman studies of salen-chxn-Ni(II) and salen-chxn-Cu(II).

In a typical ROA experiment, a randomly polarized light passes through a chiral sample, and the chiral Raman response is measured by the small intensity difference of the back-scattered right circularly polarized light (*I_R_*) versus left circularly polarized light (*I_L_*) using the scattered circularly polarized (SCP)-ROA [60]. For systems under (near) resonance, it was reported recently that the *I_R_* − *I_L_* signals contain two important contributions: resonance ROA and eCP-Raman. eCP-Raman occurs because the randomly polarized light becomes circularly polarized when it passes through a chiral sample under resonance and is absorbed differentially. The resulting circularly polarized light interacts with the chiral solute and solvent and generates circularly polarized Raman responses (*I_R_* − *I_L_*) by both the chiral solute and (achiral) solvent. We emphasize that both chiral or achiral systems may produce eCP-Raman features, and the *I_R_* − *I_L_* responses are measured in the usual channel with a regular SCP-ROA instrument. One hallmark of eCP-Raman is the huge *I_R_—I_L_* bands of (achiral) solvent, i.e., the chirality of the chiral solute is now imprinted onto the eCP-Raman response of the achiral solvent.

**Figure 12:**
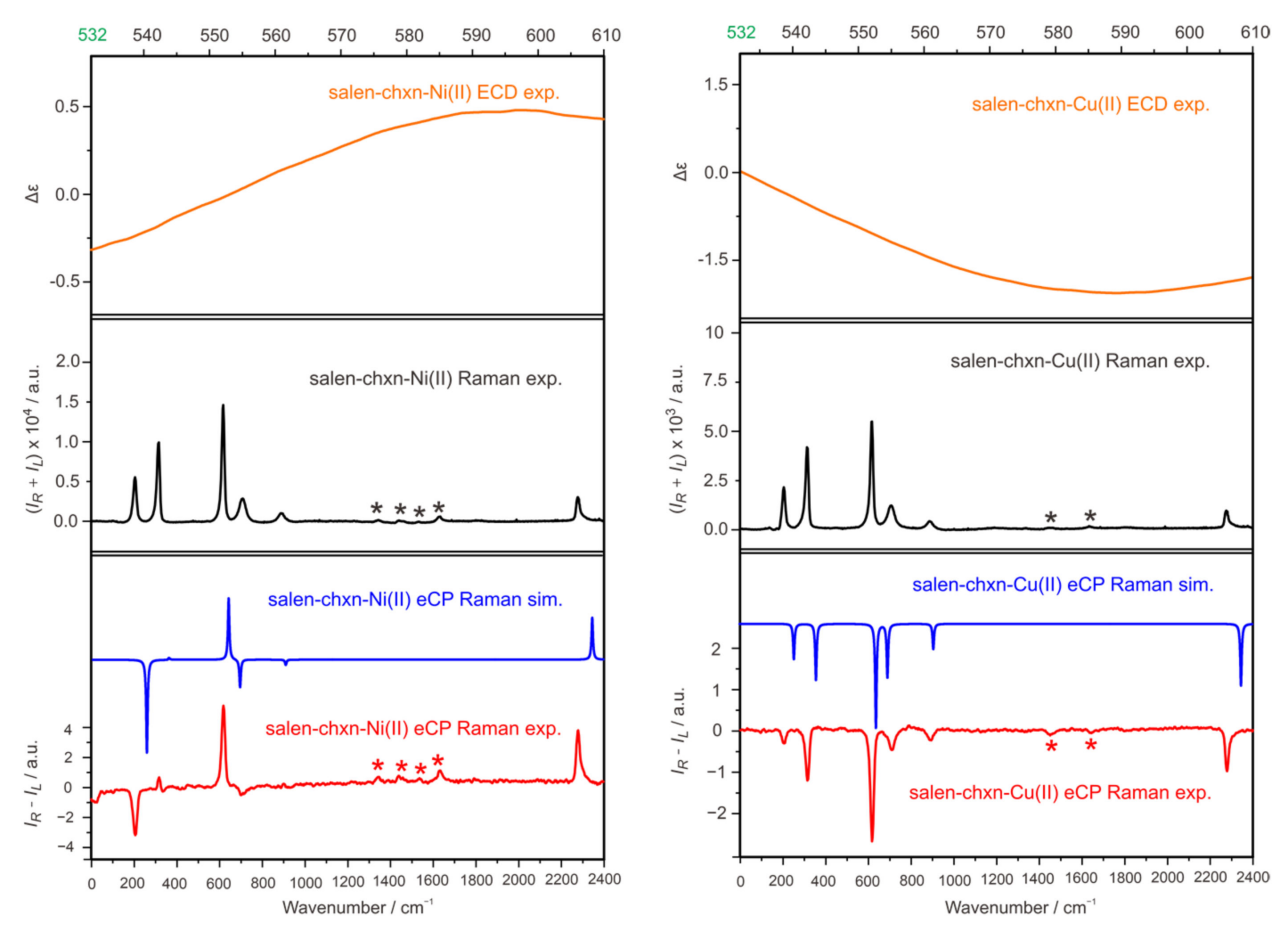
(**Top**): experimental ECD spectra from 532 nm to 610 nm of salen-chxn-Ni(II) and salen-chxn-Cu(II). (**Middle**): experimental Raman spectra of the two metal complexes. (**Bottom**): the comparison of the experimental and simulated eCP-Raman spectra of the two metal complexes. The solute bands are indicated with asterisk.

Experimental Raman and *I_R_* − *I_L_* spectra of salen-chxn-Ni(II) and salen-chxn-Cu(II) are provided in Figure 12. It is immediately obvious that both Raman and *I_R_* − *I_L_* spectra are dominated by the solvent CDCl_3_ bands, whereas the bands of the Ni(II) and Cu(II) complexes are fairly weak. Similar observations were reported in recent publications [23,24,25,61]. The huge *I_R_* − *I_L_* responses of CDCl_3_ indicate that the chiral Raman contribution is largely dominated by the eCP-Raman effect, as discussed above.

The eCP-Raman contribution can be described using the following expression [23,25]

(1) $$CID=\frac{I_{R}-I_{L}}{I_{R}+I_{L}}=\frac{ln10}{4}cL\Delta \varepsilon \left(\frac{\Delta \varepsilon^{\prime }}{\Delta \varepsilon }+DOC\right)$$

Here *CID*, the normalized circular intensity difference, is defined as (*I_R_* − *I_L_*)/(*I_R_* + *I_L_*) where *I_R_* + *I_L_* corresponds to Raman and *I_R_* − *I_L_* to eCP-Raman. ∆*ε* and ∆*ε*′ are decadic, differential absorption coefficients of the chiral solute for the excitation light at 532 nm and the scattered light at each Raman band, respectively. *C* is the concentration of the chiral solute, *L* is the optical path length, and *DOC* is the normalized degree of circularity of the vibrational transition of interest. By applying Equation (1), one can simulate eCP-Raman using *CID* times Raman.

While *c* and *L* are experimental parameters, one can, in principle, calculate ∆*ε* and ∆*ε*′ as well as *DOC*. On the other hand, theoretically, it is currently still extremely challenging to capture the ∆*ε* and ∆*ε*′ magnitudes in the weak tail regions of the ECD bands of the transition metal complexes. Therefore, we decided to utilize the experimental ECD measurements of Ni(II) and Cu(II) in the tail regions, which are included in the top panels of Figure 12. It is interesting to point out that even though the ECD intensities are very low in the region beyond 532 nm for these two complexes, the zoom-in graphs in Figure 12 show that the spectral patterns look very different in salen-chxn Ni(II) versus Cu(II). If one re-examines the *CID* equation above, it is obvious that ∆*ε* and ∆*ε*′ play a significant role in determining the signs and relative intensities of eCP-Raman bands. Therefore, one can expect drastically different eCP-Raman spectral patterns of salen-chxn-Ni(II) versus Cu(II), even though only the transition metal centers are different in these two salen-chxn metal complexes. Quantum mechanically, *DOC*, corresponding to the intensity and sign of CP-Raman signals, can be calculated using the same electric dipole–electric dipole polarizability tensors as for Raman scattering [62]. In the bottom panel of Figure 12, we compare the simulated and experimental results. As one can see, with the combined experimental and theoretical inputs, all the strong *I_R_* − *I_L_* solvent bands are fully captured using the eCP-Raman calculations, confirming the dominant nature of the light–matter interactions through the eCP-Raman mechanism.

From the above eCP-Raman discussions of the two metal complexes, one can see that the chirality information of the metal complexes is now imprinted onto the *I_R_* − *I_L_* solvent bands. These bands are generally much stronger than the corresponding ones of the metal complexes themselves because the solvent Raman bands have higher intensities than the metal complexes thanks to their large concentrations, a condition which is true in almost all solution experiments. This offers an opportunity to monitor the chirality of the complexes with only a small amount of chiral samples. By combining the high chiral sensitivity of ECD with the high-resolution capability of Raman spectroscopy, eCP-Raman is a promising new tool for absolute configuration determination and for probing excited state phenomena with the aid of improved theoretical simulations of ECD.

## 3. Materials and Methods

### 3.1. Experimental Section

The (*R*,*R*) and (*S*,*S*) salen-chxn ligands (98%) were purchased from Sigma-Aldrich, Milwaukee, USA and used without further purification. The salen-chxn-Ni(II) and -Cu(II) complexes were synthesized based on the previous literature procedures [63]. Briefly, the salen-chxn ligand was dissolved in absolute ethanol, and then the solution was heated to reflux (almost boiling). The perchlorate hexahydrate salt of Ni (or Cu) in absolute ethanol was added into the ligand solution and refluxed for 3 h. The solution mixture was concentrated using a rotavapor, and the residue was again dissolved in CH_2_Cl_2_ and ethyl acetate. The resulting solution was filtered under suction filtration to remove unreacted ligands. After that, the solvent was removed from the filtered solution using a rotavap to collect the remaining solid powder, which was further recrystallized using dichloromethane. The resulting crystals of the Ni(II) and Cu(II) complexes were collected.

All IR and VCD spectra were collected using an FTIR spectrometer (Bruker Vertex 70, Milton, Canada) coupled to a VCD model (PMA 50) [64]. The photoelastic modulator (PEM) was set at 1400 cm^−1^ for all measurements. The liquid nitrogen-cooled mercury cadmium telluride (MCT) detector was used, and the resolution was set at 4 cm^−1^. The CDCl_3_ solutions of the salen ligand and the salen-chxn-Ni(II) and salen-chxn-Cu(II) complexes were prepared in concentrations of 0.060 M, 0.060 M and 0.060 M, respectively. A demountable BaF_2_ cell was used for all measurements. For sale-chxn, salen-chxn-Ni(II) and salen-chxn-Cu (II), a 0.1 mm, 0.1 mm, and 0.05 mm Teflon spacer were used, collection time was one, one, and two hours, respectively. The final IR spectra were baseline corrected by the subtraction of the solvent spectrum measured under the same conditions, while the VCD spectra were obtained using the usual (R-S)/2 method.

The UV-Vis and ECD spectra of the salen-chxn ligand in acetonitrile were measured using a JASCO-810 ECD spectrometer with a concentration of 1 mM and a path length of 0.2 mm. The UV-Vis and ECD spectra of the salen-chxn-Ni(II) and -Cu(II) complexes in acetonitrile were collected using a Jasco-1700 spectrometer. The concentration was 1 mM (5 mM), and the path length of sample cell was 1 mm.

The mass spectrometry and NMR data of the two metal complexes are reported in Point S1, Supplementary Materials. The expected mass weights of the two complexes were confirmed, and the NMR data are consistent, with the Ni(II) complex being diamagnetic and the Cu(II) complex being paramagnetic with broad NMR lines. The raw VCD and ECD spectra of the (*R*,*R*) and (*S*,*S*)-salen-chxn-Ni(II) and -Cu(II) complexes studies are also presented in Point S1.

The eCP-Raman spectra were measured using a Biotools (Jupiter, USA) chiral Raman spectrometer in the backward scattered circular polarized (SCP) scheme. The collection time for salen-chxn-Ni(II) and salen-chxn-Cu(II) was 5 and 25 h, respectively. CDCl_3_ was used as the solvent, and the concentration was 10 mM and 5 mM for the Ni(II) and Cu(II) complexes, respectively.

### 3.2. Theoretical

To systematically explore the possible conformers of the ligand and the complexes, we utilized the CREST code [29], including the generalized Born (GB) based GBSA implicit solvation model, using CDCl_3_ as the solvent. Built upon the previous semiempirical tight-binding (TB) quantum chemistry method, GFN-xTB [65], the CREST code offers fast and reliable exploration and screening of the conformational space of mid- to large-sized molecules. A multitiered approach developed before [38] was applied to ensure fast and complete conformational searches. This includes several steps: (1) use CREST to generate candidates; (2) apply DFT optimizations of the CREST candidates with relaxed convergence criteria at the revPBE-D3/def2-SVP [47] level, with the empirical D3 dispersion correction; (3) do a single-point energy evaluation at the B3LYP-D3/def2-TZVP level of the optimized structures in step (2); Step 2 and (3) were done using Molpro [66]; (4) perform the final geometry optimizations and harmonic frequency calculations using the Gaussian 16 package [67].

In the current study, the step (4) calculations were carried out at the B3LYP-D3BJ/def2-TZVP level for the ligand, at the B3LYP/6-311++G(d,p) level and the B3LYP-D3BJ/def2-TZVP (for all atoms except Ni and Cu) + LANL2DZ(for Ni and Cu) for the metal complexes. To account for the bulk solvent environment, the implicit solvent (ε = 4.81 for CDCl_3_) was included using the integral equation formalism (IEF) version of the PCM [48]. A Lorentzian band shape with a half-width at half-height (HWHH) of 4 cm^−1^ was applied to the simulations of IR and VCD spectra. In order to improve the frequency accuracy of the simulated spectra, a linear correlation method, as described in [68], was utilized to scale the simulated frequencies in the current study.

The TDDFT calculations were employed to calculate the excited state energies, oscillator strength and optical rotation dispersion calculations at the B3LYP-D3BJ/6-311++G (d,p) and MN12L/6-311++G(d,p) levels of theory with the PCM of acetonitrile (ε = 35.688). The first 200 and 250 electronic states were taken into account for the ligand and the two metal complexes in simulating their UV-Vis and ECD spectra, respectively. A Gaussian line shape with an HWHH of 0.15 eV was used for the simulations of UV−Vis and ECD spectra.

## 4. Conclusions

The stereochemistry properties of a flexible salen-chxn ligand and two salen transition metal complexes, salen-chxn-Ni(II) and salen-chxn-Cu(II), were investigated using a combined experimental and theoretical approach with a series of chiroptical tools: UV-Vis and ECD, IR and VCD, and Raman and eCP-Raman. While many conformational candidates were generated with the systematic CREST conformational searches, the main low-energy conformations for the three molecular systems studied are associated with the different tBu arrangements. The excellent agreement between the experimental and simulated IR and VCD spectra of salen-chxn demonstrated that there are three main conformational species in solution with an estimated abundance of 60% salen-chxn-III and 20% each of salen-chxn-I and -II, also supported by the UV-Vis and ECD study. The complexation with Ni(II) and Cu(II) leads to a strong preference for the staggered–staggered ligand conformation with a nearly squared planar coordination geometry and, at the same time, induces an absolute configuration of the metal center, i.e., the helicity of the complexes. We were able to confirm the structural preference and determine the helicity of the metal complexes to be M for (*R*,*R*)-salen-chxn ligand based on the good agreement between the observed and simulated chiroptical spectra. We also showed that the salen-chxn-Ni(II) and -Cu(II) are under (near) resonance with the Raman laser at 532 nm and measured the corresponding eCP-Raman spectra of them, which are dominated by the induced solvent chiral Raman bands. These observed chiral Raman solvent bands agree well with the simulated ones, demonstrating that the mechanism is well understood and highlighting eCP-Raman as a promising new tool for absolute configuration determination. Moreover, we extended the application of the exciton chirality method to the metal complexes and extracted their helicity information from the observed VCD spectra. Interestingly, it was found that the excitation chirality method did not work for the ECD interpretation of all three systems. Using the calculation results, we highlighted the important criteria for properly applying the excitation chirality method and discussed the reasons for its failure in the case of ECD interpretation of the three compounds studied. The current work demonstrates the advantages of applying multiple chiroptical spectroscopic techniques in a combination of theoretical modelling in order to explore conformational landscapes, ligand chirality, and helicity of transition metal complexes in solution.

## Data Availability

Data is contained within the article.

## Supplementary Materials

The following supporting information can be downloaded at: https://www.mdpi.com/article/10.3390/molecules28062571/s1. Figure S1: QTAIM analyses of salen-chxn-I and -III; Figure S2: Simulated UV-Vis and ECD spectra of three salen-chxn ligand conformers at the MN12L/def2-TZVP level; Figure S3: Simulated IR and VCD spectra of the three conformers of salen-chxn-Ni(II) and salen-chxn-Cu(II); Figure S4: Definition of the helicity angle; Figure S5: Simulated UV-Vis and ECD spectra of salen-chxn-Ni(II) and salen-chxn-Cu(II) with the detailed contributions of individual electronic transitions indicated by sticks. Point S1: The mass spectrometry, NMR, raw VCD and ECD data of salen-chxn-Ni(II) and -Cu(II).

## Sample Availability

Samples of the compounds are available commercially or can be made following the previous literature.
