# Supplementary material for: Stereochemical Properties of Two Schiff-Base Transition Metal Complexes and Their Ligand by Using Multiple Chiroptical Spectroscopic Tools and DFT Calculations
Source: Molecules. 2023 Mar 12;28(6):2571. doi: 10.3390/molecules28062571 (PMC10054095; doi:10.3390/molecules28062571)

Supplementary Material

Stereochemical Properties of Two Schiff-Base Transition Metal Complexes and Their Ligand by Using Multiple Chiroptical Spectroscopic Tools and DFT Calculations

Guojie Li [1], Dan Li [1,2], Mutasem Alshalalfeh [1], Joseph Cheramy [1], Hui Zhang [2] and Yunjie Xu [1,*]

1 Department of Chemistry, University of Alberta, Edmonton, AB T6G 2G2, Canada

2 Department of Chemistry, College of Chemistry and Chemical Engineering, Xiamen University, Xiamen 361005, China

* Correspondence: yunjie.xu@ualberta.ca; Tel.: +1-780-492-1244

Contents

Figure S1. QTAIM analyses of salen-chxn-I and -III with their bond critical points indicated S2

Figure S2. Simulated UV-Vis and ECD spectra of the three salen-chxn ligand conformers in acetonitrile solution at the MN12L/def2-TZVP level. S2

Figure S3. Simulated IR and VCD spectra of the three conformers of salen-chxn-Ni(II) and salen-chxn-Cu(II)............ S3

Figure S4. Definition of the metal helicity S3

Figure S5. Simulated UV-Vis and ECD spectra of salen-chxn-Ni(II) and salen-chxn-Cu(II) with the detailed contributions of individual electronic transitions indicated S4

Point S1. The mass spectrometry, NMR, raw VCD and ECD data of salen-chxn-Ni(II) and -Cu(II)……………………..S4

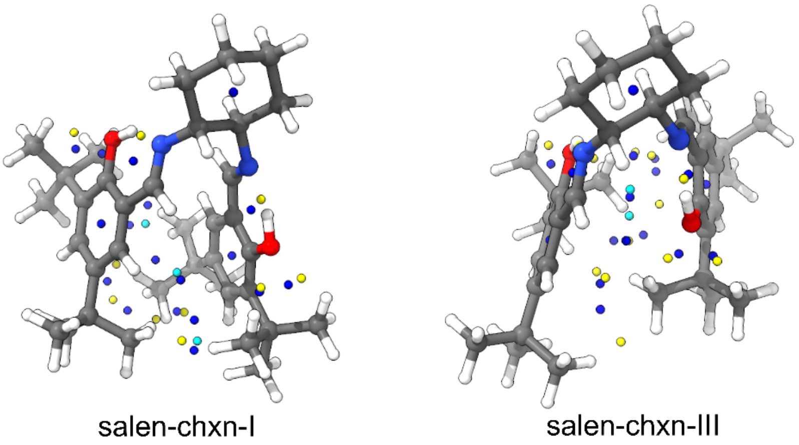

Figure S1. The QTAIM analysis of salen-chxn-I and -III with their bond critical points indicated: yellow = bond critical point, blue = ring critical point, and cyan = cage critical points. Salen-chxn-I has one more cage critical point than -III, stabilizing its geometry.

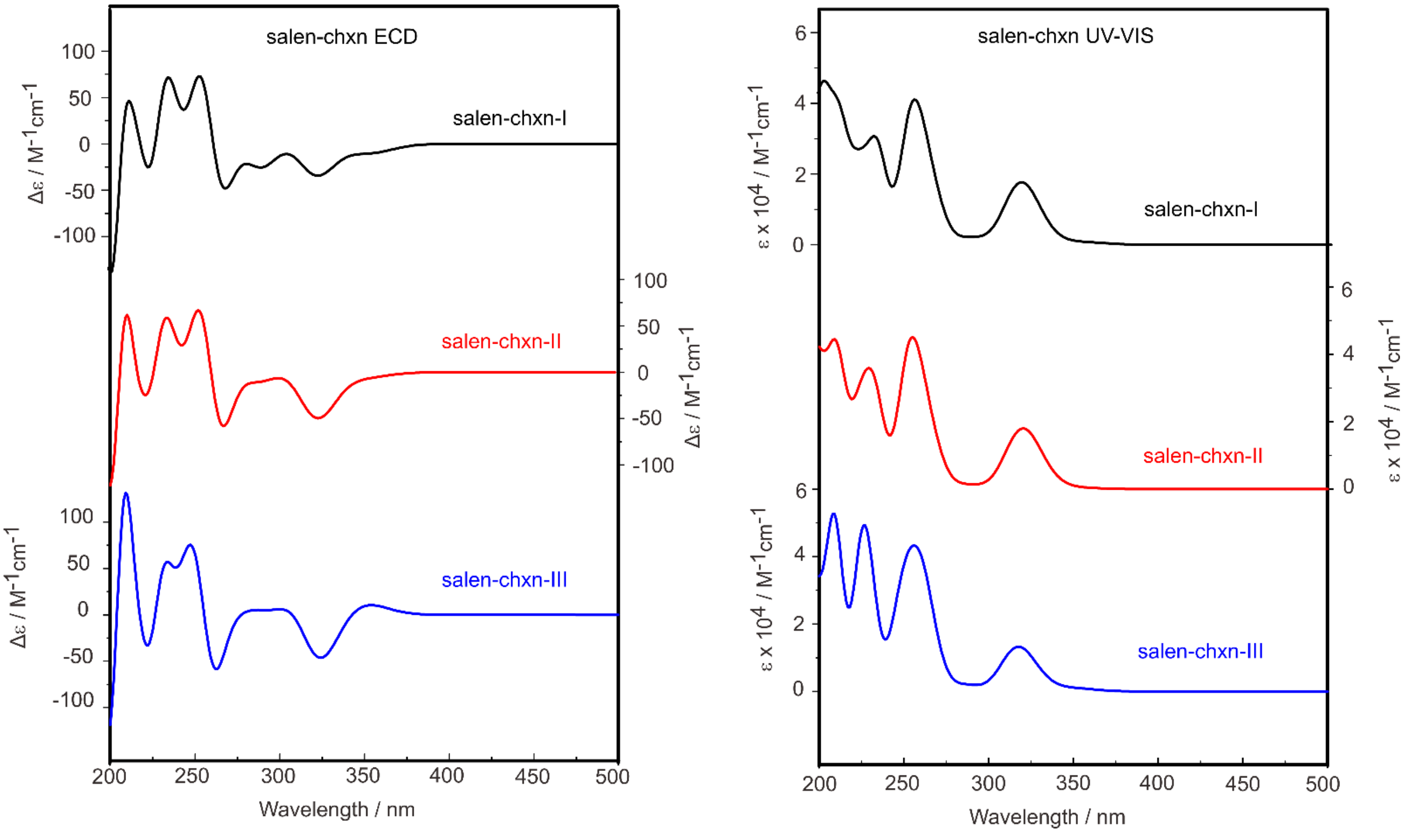

Figure S2. The theoretical UV-Vis and ECD spectra of three salen-chxn ligand conformers in acetonitrile solution at the MN12L/def2-TZVP level. The first 200 electronic states were included in the calculations.

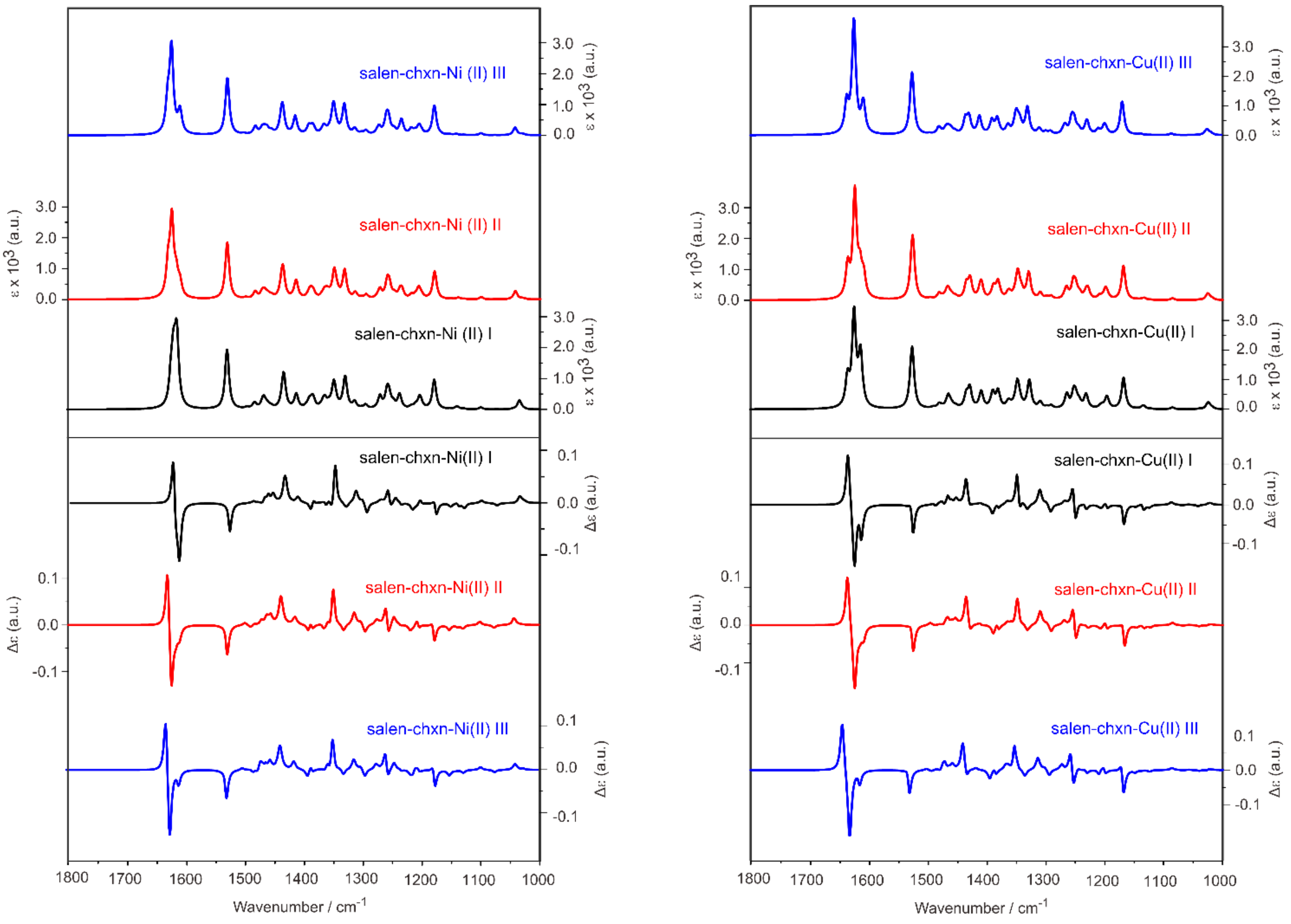

Figure S3. The simulated IR (top) and VCD (bottom) spectra of the three conformers of salen-chxn-Ni(II) and salen-chxn-Cu(II) at the B3LYP-D3BJ/6-311++G(d,p) level with the PCM of chloroform.

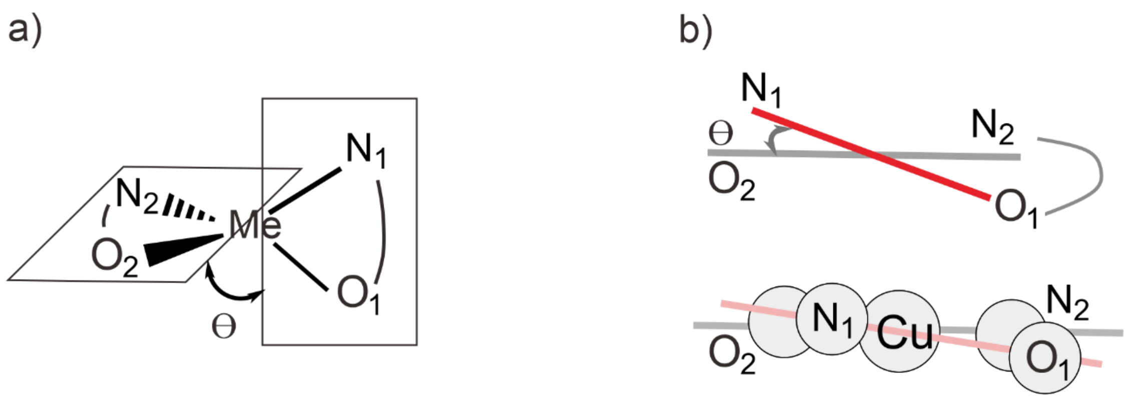

Figure S4. (a) Definition of the helicity-determining angle θ. For square planar, θ=0°, whereas θ=90° for tetrahedral. N_1 and O_1 are atoms of one aromatic branch, and N_2 and O_2 are atoms of the other branch. (b) Illustration of the *M*-helicity in the near square planar metal complexes with the (R,R) ligand, viewing the coordination arrangement in (a) from the right side. Note that N_2 and N_4 are connected by the cyclohexane ring. We use (R,R)-salen-chxn-Cu(II) as an example. Note that all other atoms are removed for clarity.

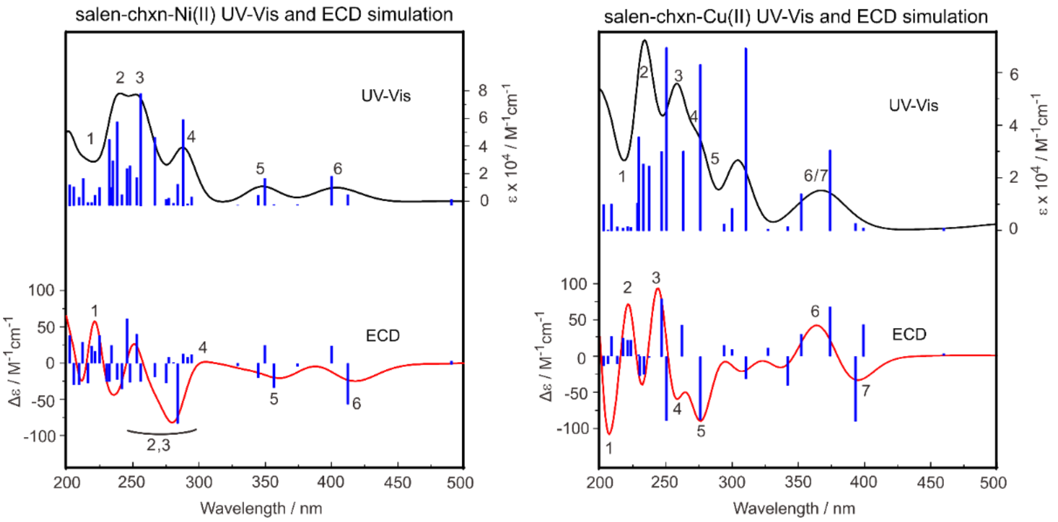

Figure S5. The simulated UV-Vis (top) and ECD (bottom) spectra of salen-chxn-Ni(II) and salen-chxn-Cu(II) with the detailed contributions of individual electronic transitions indicated by sticks. The calculations were done at the B3LYP-D3BJ/6-311++G(d,p) and 250 electronic states were included in the calculation.

Point S1. (a) The mass spectrometry and NMR data of salen-chxn-Ni(II) and -Cu(II).

salen-chxn-Ni(II): TOS MS: m/z 603.3 $(M+H)^+$. ^{1}H NMR (s400 MHz, $CDCl_3$, 25^{0}C, TMS): δ 1.26 (s,18H, tBu), δ 1.31 (d,4H, cyclohexane), δ 1.42 (s,18H, tBu), δ 1.88 (s,2H, CH_2-cyclohexane), δ 2.42 (s,2H, CH_2-cyclohexane), δ 3.04 (s,2H, CH-cyclohexane), δ 6.87 (d,2H, Benzene H), δ 7.30 (d,2H, Benzene H), δ 7.39 (s,2H, imine H, N=C-H).

salen-chxn-Cu(II): TOS MS: m/z 608.3 $(M+H)^+$. ^{1}H NMR (s400 MHz, $CDCl_3$, 25^{0}C, TMS): δ 1.06 (s,18H, tBu), δ 1.65 (d,4H, cyclohexane), δ 2.03 (s,18H, tBu), δ 2.17 (s,2H, CH_2-cyclohexane), δ 7.25 (d,2H, Benzene H), δ 8.04 (s,2H, imine H, N=C-H).

(b) The raw experimental VCD (top) and ECD (bottom) spectra of (R,R) and (S,S)-salen-chxn-Ni(II) and -Cu(II).

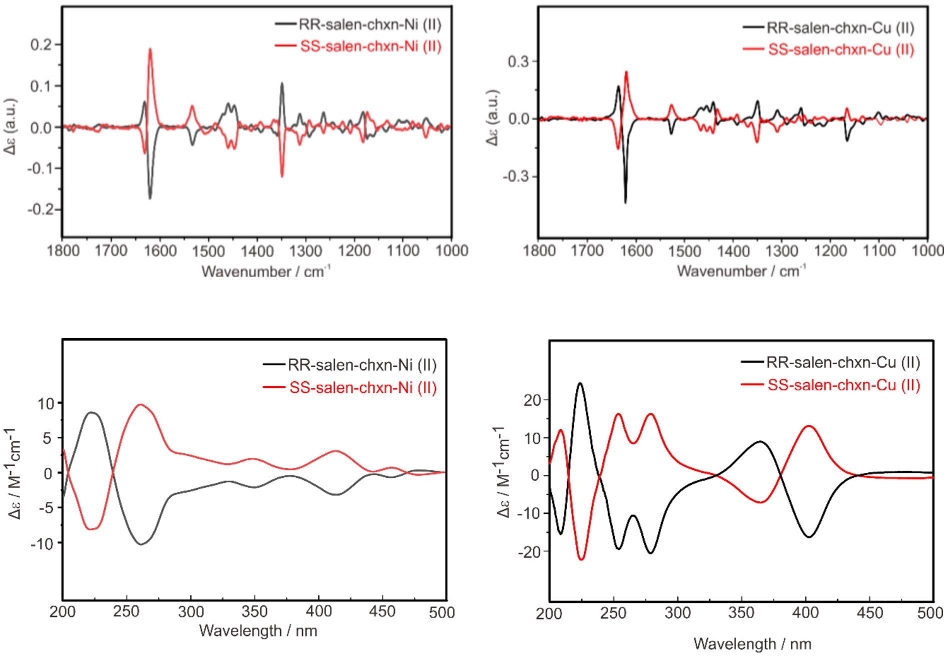

Supplement: Supplementary file 1 [file molecules-28-02571-s001.zip › molecules-2250989-supplementary.pdf]
